# Thermal Decomposition of Maya Blue: Extraction of Indigo Thermal Decomposition Steps from a Multistep Heterogeneous Reaction Using a Kinetic Deconvolution Analysis

**DOI:** 10.3390/molecules24132515

**Published:** 2019-07-09

**Authors:** Yui Yamamoto, Nobuyoshi Koga

**Affiliations:** Chemistry Laboratory, Department of Science Education, Graduate School of Education, Hiroshima University, 1-1-1 Kagamiyama, Higashi-Hiroshima 739-8524, Japan

**Keywords:** Maya blue, indigo, palygorskite, sepiolite, thermal decomposition, kinetic deconvolution analysis

## Abstract

Examining the kinetics of solids’ thermal decomposition with multiple overlapping steps is of growing interest in many fields, including materials science and engineering. Despite the difficulty of describing the kinetics for complex reaction processes constrained by physico-geometrical features, the kinetic deconvolution analysis (KDA) based on a cumulative kinetic equation is one practical method of obtaining the fundamental information needed to interpret detailed kinetic features. This article reports the application of KDA to thermal decomposition of clay minerals and indigo–clay mineral hybrid compounds, known as Maya blue, from ancient Mayan civilization. Maya blue samples were prepared by heating solid mixtures of indigo and clay minerals (palygorskite and sepiolite), followed by purification. The multistep thermal decomposition processes of the clay minerals and Maya blue samples were analyzed kinetically in a stepwise manner through preliminary kinetic analyses based on a conventional isoconversional method and mathematical peak deconvolution to finally attain the KDA. By comparing the results of KDA for the thermal decomposition processes of the clay minerals and the Maya blue samples, information about the thermal decomposition steps of the indigo incorporated into the Maya blue samples was extracted. The thermal stability of Maya blue samples was interpreted through the kinetic characterization of the extracted indigo decomposition steps.

## 1. Introduction

Thermal decomposition of inorganic solids is a complex heterogeneous reaction that is regulated by chemical kinetics and physio-geometrical constraints [1,2,3]. In addition, consecutive or concurrent reaction steps that originated from both chemical [4,5,6,7,8,9] and physio-geometrical reaction mechanisms can occur [10,11,12,13,14,15,16], wherein the individual reaction steps may be kinetically dependent on one another. For a multistep process in a homogeneous system, the kinetic behavior can be formalized using concentrations of reactant and intermediate and each reaction step can be verified according to probability considerations. By contrast, in the heterogeneous system, the rigorous formalization of the kinetic equation for multistep reactions is not so easy because of the physico-geometrical constraints of each reaction step and these complex interactions [17]. When each reaction step that exhibits Arrhenius-type temperature dependence can be approximated to be kinetically independent from the other steps, kinetic deconvolution analysis (KDA) can be applied to find an empirical solution for the kinetic description of multistep solid-state reactions [18,19], as follows:

(1) $$\frac{d\alpha }{dt}=\overset{N}{\underset{i=1}{\sum }}c_{i}A_{i}\exp\left(-\frac{E_{a,i}}{RT}\right)f_{i}\left(\alpha_{i}\right)\;\mathrm{with}\;\overset{N}{\underset{i=1}{\sum }}c_{i}=1\;\mathrm{and}\;\overset{N}{\underset{i=1}{\sum }}c_{i}\alpha_{i}=\alpha$$

where *α*, *c*, *A*, *E*_a_, and *R* are the fractional reaction, contribution, Arrhenius pre-exponential factor, apparent activation energy, and the gas constant, respectively. The subscript *i* denotes a reaction step out of a total *N* steps. The function *f*(*α*) describes the physico-geometrical reaction mechanism as formalized by considering the rate-limiting step of the reaction and the reaction geometry [1,2,3]. Despite the empirical nature of the kinetic analysis using KDA, the results provide necessary information to gain further insights into consecutive or concurrent kinetic features [20,21,22], as well as practically useful information about the multistep process, including the contribution (*c_i_*) and apparent kinetic parameters (*A_i_*, *E*_a,*i*_, and *f_i_*(*α_i_*)) of each reaction step *i*. Using the results of KDA, the overall reaction process, under a specific heating condition, can be reproduced or simulated. By comparing the results of KDA among a series of samples and under different reaction conditions, characteristics of multistep kinetic behavior can be correlated to different components of a composite sample [23,24,25,26] and specific reaction conditions [27,28]. KDA is also used to extract kinetic information about a selected reaction step from the overall process [29,30].

A multistep heterogeneous thermal decomposition can be observed for inorganic–organic hybrid materials. One example of such a material is Maya blue (MB), a well-known pigment used in the Mayan civilization. MB is a hybrid compound of a microporous clay mineral and indigo [31,32,33]. Palygorskite and sepiolite are the typical clay minerals used in the preparation of MB. These fibrous clay minerals exhibit external and internal nanochannels, which are typically filled with zeolitic water [34,35,36,37,38,39,40,41]. MB is produced by the replacement of the zeolitic water with indigo molecules [31,34,35,36]. Notably, MB exhibits high stabilities against thermal treatment, light exposure, and acid and/or base attacks. Consequently, the structural characteristics of MB have been intensively studied using spectroscopic techniques that include Fourier transform infrared (FT-IR) and Raman spectroscopies [31,34,35,41,42,43,44]. The thermal behavior of MB has also been studied using thermoanalytical techniques [34,36,42]. The formation of strong hydrogen bonds between the structural water of the clay mineral and the carbonyl and amino functional groups of the indigo molecules was reported as a possible reason for the high stabilities of MB [37]. MB with palygorskite as the clay mineral is expected to be more stable compared to MB with uses sepiolite, because of the higher number of hydrogen bonds available to form between the indigo and the substrate mineral [35]. It was also reported that the indigo molecules incorporated into the clay mineral substrate transform into dehydroindigo during the heating process used to prepare MB [37,43,45]. The thermal decomposition of MB begins just above room temperature and indicates partially overlapping multistep processes upon further heating, which may be composed of dehydration steps of zeolitic, coordinating, and structural waters; decomposition of hydroxides in the substrate mineral; and sublimation/decomposition of indigo molecules [34,36,42]. Indigo molecules that have been incorporated into the clay mineral are known to have a higher thermal stability, as confirmed by thermoanalytical curves, compared to pure indigo crystals [34].

Kinetic characterization of the thermal decomposition of MB is a promising approach to evaluating its thermal stability. However, its complex multistep thermal behavior interferes with a successful and straight-forward kinetic analysis. Application of KDA to the thermal decomposition of MB is one possible empirical method of separating the overlapping reaction steps and kinetically characterizing each one to determine the reaction steps that relate specifically to the thermal decomposition of indigo molecules. In the present study, MBs based on palygorskite (P-MB) and sepiolite (S-MB) were prepared by heating solid mixtures of indigo and clay minerals. The thermal decomposition processes of the purified clay mineral substrate samples and the MB samples were analyzed kinetically using KDA, after the necessary preliminary kinetic approaches. By comparing the kinetic results for the thermal decomposition of MB and its clay mineral substrates, the thermal decomposition steps of the indigo molecules were extracted. Using the kinetic information for the thermal decomposition steps of the indigo molecules, the kinetic stabilities of the indigo molecules incorporated in different clay mineral matrices were compared to one another and those of pure indigo crystals.

## 2. Results and Discussion

### 2.1. Sample Preparation and Characterization

**Figure 1:**
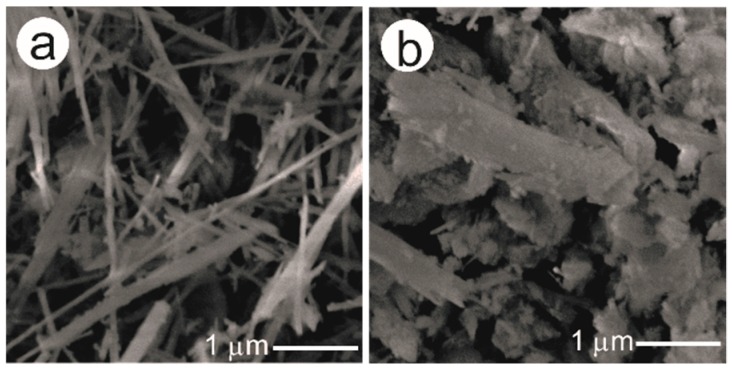
SEM images of the clay minerals after treatment with HCl(aq): (**a**) Palygorskite and (**b**) sepiolite.

The characterization details of the purchased palygorskite and sepiolite samples are described in Section S1(1) in the Supplementary Materials. Since a CaCO_3_ impurity was found in both clay mineral samples, they were purified using HCl(aq) before use. Figure 1 represents the scanning electron microscope (SEM) images of the clay minerals after treatment with HCl(aq). The palygorskite sample is an agglomerate of needle-like crystal with a length of approximately 2–5 μm (Figure 1a). Agglomerates of columnar crystals, with a length of approximately 1–2 μm, are characteristic of the sepiolite sample (Figure 1b).

**Figure 2:**
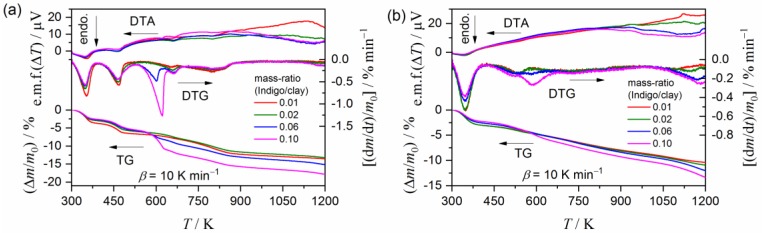
TG–DTG–DTA curves for the heat-treated indigo/clay mineral mixtures synthesized using different initial ratios: (**a**) Indigo/palygorskite and (**b**) indigo/sepiolite.

The coloration changes in the indigo–clay mineral mixture before and after heating are described in Section S1(2) in the Supplementary Materials. Figure 2 compares thermogravimetry (TG)–derivative thermogravimetry (DTG)–differential thermal analysis (DTA) curves for the heat-treated samples with different indigo/clay mineral ratios. The major difference between the samples with different indigo/clay mineral ratios is the mass-loss step initiated at approximately 525 K and 535 K for the palygorskite and sepiolite substrate samples, respectively, in which the mass-loss value and the DTG peak height increase with an increasing amount of indigo. The temperature range of the mass-loss step agrees with that for the sublimation/decomposition of pure indigo crystals (Figure S9). Therefore, it is likely that, in the samples with higher indigo/clay mineral ratios, excess indigo remains unreacted with the clay mineral substrates.

**Figure 3:**
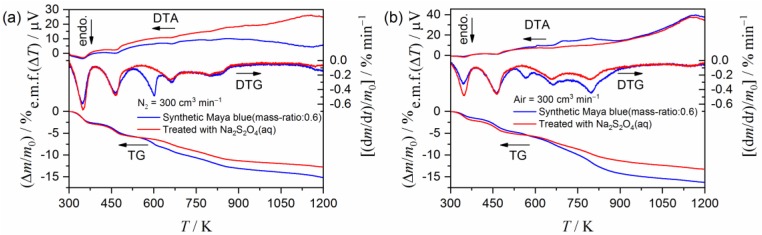
Comparison of the TG–DTG–DTA curves for the heat-treated indigo/palygorskite samples before and after treatment with Na_2_S_2_O_4_(aq) recorded in (**a**) flowing N_2_ and (**b**) flowing air.

Figure 3 compares the TG–DTG–DTA curves for the heat-treated indigo/palygorskite sample and those further treated with Na_2_S_2_O_4_(aq) for removing excess indigo. The TG–DTG–DTA curves were recorded under an atmosphere of flowing N_2_ (Figure 3a) and clearly indicate that the mass-loss step initiated at approximately 525 K disappears after samples are treated with Na_2_S_2_O_4_(aq). For the sample treated with Na_2_S_2_O_4_(aq), several mass-loss steps that occur at the higher temperatures also disappeared in the TG–DTG–DTA curves recorded in flowing air (Figure 3b). The disappeared mass-loss steps are expected to be attributed to either oxidation or combustion of the thermal decomposition product of indigo. The comparable results of Na_2_S_2_O_4_(aq) treatment were also observed for the heat-treated indigo/sepiolite samples, as shown in Figure S10. These results indicate that the removal of excess indigo from the MB samples was successful. Figure S11 compares the sample coloration before and after the Na_2_S_2_O_4_(aq) treatment. The faded color that results after the treatment arises from the removal of excess indigo. The color fading is characterized by a decrease in the ultraviolet-visible (UV-Vis) absorption in the wavelength range of 425–600 nm and the appearance of a maximum absorption at approximately 650 nm, as illustrated in Figure S12. Figure S13 presents the SEM images of the samples treated with Na_2_S_2_O_4_(aq). The appearance of the synthesized MB samples was not significantly different from those of the palygorskite and sepiolite samples (Figure 1).

### 2.2. Kinetic Analysis of the Thermal Decomposition of Clay Minerals

**Figure 4:**
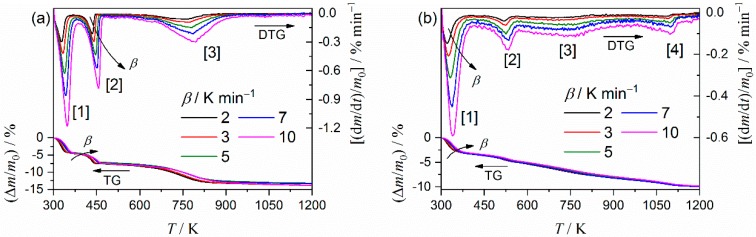
TG–DTG curves for the thermal decomposition of the purified clay minerals recorded at different *β* in a flow of N_2_ gas: (**a**) Palygorskite and (**b**) sepiolite.

Figure 4 presents TG–DTG curves for the purified clay minerals, recorded at different heating rates (*β*) in the flow of N_2_ gas. The thermal decomposition processes of both clay minerals are multistep processes comprised of three and four distinguishable DTG peaks for palygorskite (Figure 4a) and sepiolite (Figure 4b), respectively. The systematic shift of all the distinguishable DTG peaks to higher temperatures with increasing *β* was observed for both samples. This is a normal feature for the kinetic process. The average value for the total mass loss during heating the samples to 1223 K was 13.6 ± 0.3% and 9.9 ± 0.1% for palygorskite and sepiolite, respectively. The overall thermal decomposition behaviors of palygorskite and sepiolite approximately agree with those previously reported [34,46,47]. The first to third DTG peaks in both the samples correspond to the thermal dehydration of zeolite water, the first coordinated water, and the second coordinated water. The fourth DTG peak in the thermal decomposition of sepiolite is attributed to the thermal dehydration of structural water.

As part of the preliminary kinetic approach to the multistep thermal decomposition process, the isoconversional kinetic analysis was examined for the overall thermal decomposition. For the ideal single-step reaction, Equation (2) can be used as the fundamental kinetic equation [48].

(2) $$\frac{d\alpha }{dt}=A\exp\left(-\frac{E_{a}}{RT}\right)f\left(\alpha \right)$$

Taking the natural logarithm of both sides of Equation (2), one can obtain the following equation:

(3) $$\ln\left(\frac{d\alpha }{dt}\right)=\ln\left[Af\left(\alpha \right)\right]-\frac{E_{a}}{RT}$$

**Figure 5:**
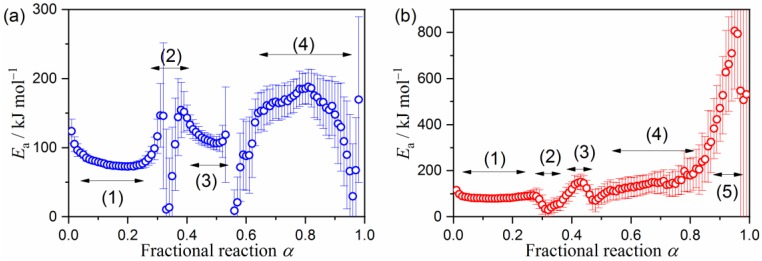
The apparent *E*_a_ values at different *α* with reference to the total mass-loss during the overall thermal decomposition of the purified clay minerals, determined by the Friedman plot: (**a**) Palygorskite and (**b**) sepiolite.

At the selected α, the plot of the left-hand side of Equation (3) versus the reciprocal temperature should exhibit a linear correlation when the value of ln[*Af*(*α*)] is constant. The apparent *E*_a_ values at different *α* can be calculated from the slope of the plot, known as a Friedman plot [49]. The application of the Friedman plot to the overall kinetic data of the multistep thermal decomposition process recorded at different *β* is not supported by theory, because more than one reaction step overlaps at each *α*. Even so, some possibility of finding α region characterized by a relevant *E*_a_ values or a specific trend of the *E*_a_ variation is still anticipated. Figure 5 illustrates the *E*_a_ values at different *α* for the overall thermal decomposition. For the thermal decomposition of palygorskite, four distinguishable reaction steps are expected from the constant *E*_a_ regions and the region that exhibits specific *E*_a_ variation trends (Figure 5a). The regions assigned as (1), (3), and (4) correspond to the major reaction steps observed as distinguishable DTG peaks. Five distinguishable α regions were found for the thermal decomposition of sepiolite (Figure 5b), in which the regions assigned as (1), (3), (4), and (5) correspond to the major DTG peaks.

Based on the results of the empirical application of the isoconversional method to the multistep thermal decomposition process, the component reaction steps were deconvolved based on the DTG curves by mathematical deconvolution analysis (MDA); that is, the statistical shape analysis assuming overlapping independent peaks are present [18,50,51].

(4) $$\frac{dm}{dt}=\overset{N}{\underset{i=1}{\sum }}F_{i}\left(t\right)$$

In Equation (4), *N* is the number of component peaks. The value *F_i_*(*t*) is the statistical function used to satisfactorily fit the component peak *i*. As the component DTG peaks typically have an asymmetric shape, one of the statistical functions that are applicable to symmetric peaks, such as the Weibull and Frazer–Suzuki functions, is favorable for MDA [18,50,51]. According to the number of distinguishable regions of *α* observed in the results of the isoconversional kinetic analysis (Figure 5), MDA for the thermal decomposition of palygorskite and sepiolite was carried out by setting *N* = 4 and *N* = 5, respectively. The Weibull function (Equation (S1)) was applied to fit all the component peaks.

**Figure 6:**
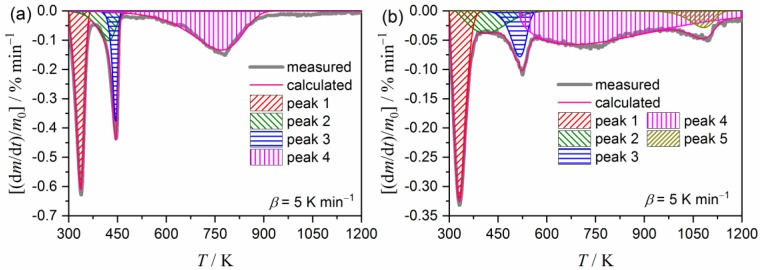
Typical results of MDA applied to the multistep thermal decomposition of the clay minerals: (**a**) Palygorskite and (**b**) sepiolite.

Figure 6 illustrates a typical result of the MDA. The second DTG peaks in both samples are described by the partial overlapping of two peaks in the MDA results. The primary outcome from the MDA is the rough estimation of the contribution *c_i_* of each reaction step *i* with reference to the overall reaction. Table S1 lists the contribution of each component step *i*. In both the samples, the first and fourth deconvolved steps, which correspond to the dehydration of zeolite water and the second coordinated water, have been indicated as significant contributions to the overall thermal decomposition.

The other outcome from MDA is the separated kinetic curves for each reaction step. The features of the separated kinetic curves and the formal kinetic analyses for these kinetic curves are described in Section S2 in the Supplementary Materials.

Based on the results obtained by the preliminary kinetic approaches using the conventional isoconversional method and MDA, the overall kinetic curves were analyzed by assuming that the overlapping multistep process was comprised of independent reaction steps. In this case, the cumulative kinetic equation in Equation (1) is applicable [17,18,19]. For the kinetic model function *f_i_*(*α_i_*) for each reaction step *i*, an empirical kinetic model that accommodates different types of the physico-geometrical reaction mechanisms and those that deviate are needed to obtain the sophisticated fit for the calculated kinetic curve to the experimental kinetic curve. The Šesták–Berggren (SB) model with three kinetic exponents [52,53,54], SB(*m*, *n*, *p*), is one such empirical kinetic model with the high flexibility needed for the fitting, as follows:

(5) $$f\left(\alpha \right)=\alpha^{m}{\left(1-\alpha \right)}^{n}{\left[-\ln\left(1-\alpha \right)\right]}^{p}$$

The nonlinear least squares analysis for fitting the calculated kinetic curve to the calculated kinetic curve while simultaneously optimizing all the kinetic parameters in Equations (1) and (5) is a typical procedure of KDA [17,18,19]. For reliable KDA, appropriate initial values of all the kinetic parameters that will be optimized through KDA are necessary. For the thermal decomposition of palygorskite and sepiolite, the initial *c_i_* and *E*_a,*i*_ values were adapted from the results of the MDA (Table S1). The initial kinetic exponents in the SB model were set to SB(0, 1, 0), which is the first-order kinetic model. Then, the order of *A_i_* values was determined graphically by monitoring the fit of the calculated kinetic curve to the experimental kinetic curve. After inputting all the initial values, KDA was run to optimize the values through nonlinear least squares analysis to minimize the sum of squares of the differences between the experimental and calculated kinetic curves, as follows:

(6) $$F=\overset{M}{\underset{j=1}{\sum }}{\left[{\left(\frac{d\alpha }{dt}\right)}_{\exp,j}-{\left(\frac{d\alpha }{dt}\right)}_{\mathrm{cal},j}\right]}^{2}$$

where *M* is the total number of data points in the experimental kinetic curve at a *β* value.

**Figure 7:**
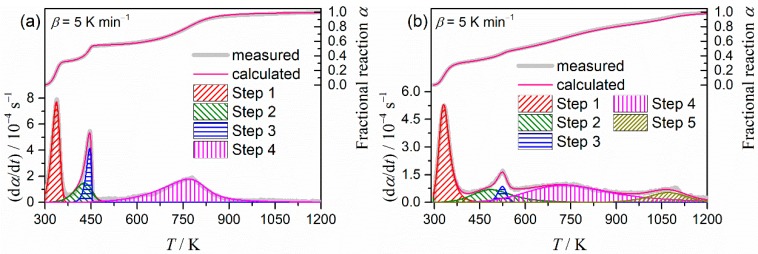
Typical results of KDA for the thermal decomposition of the purified clay minerals: (**a**) Palygorskite and (**b**) sepiolite.

**Figure 8:**
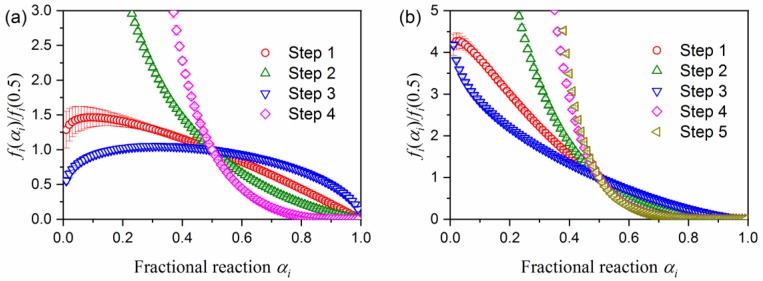
Rate behaviors for each reaction step of the thermal decomposition of (**a**) palygorskite and (**b**) sepiolite, reproduced from SB (*m_i_*,*n_i_*,*p_i_*) optimized by KDA.

Figure 7 illustrates typical results of the KDA for the thermal decomposition of the purified palygorskite (Figure 7a) and sepiolite (Figure 7b). Regardless of the kinetic curve recorded at different *β* values, the calculated kinetic curve was fit to the experimental kinetic curve with a determination coefficient for the nonlinear least squares analysis (*R*^2^) better than 0.99. The optimized kinetic parameters for each reaction step for the thermal decompositions of the purified palygorskite and sepiolite are summarized in Table 1. The contributions for the first reaction step, attributed to the thermal dehydration of zeolite water, were comparable between the two samples. This was also true for the thermal dehydration of the second coordinated water, which appeared as the fourth reaction step for the thermal decompositions of palygorskite and sepiolite. The optimized *E*_a_ values for each reaction step did not significantly change from the initial values in both samples. The rate behavior of each reaction step was simulated from the SB(*m_i_*, *n_i_*, *p_i_*) model with the optimized kinetic exponents, as illustrated in Figure 8. The first reaction step, i.e., the thermal dehydration of zeolite water, exhibits nearly linear deceleration in both samples. The linear deceleration behavior was also seen for the third reaction step of the thermal decomposition of sepiolite. The corresponding reaction step for the thermal dehydration of the first coordinated water in the thermal decomposition of palygorskite exhibited zero-order-like behavior in a wide *α*_3_ range. For the other reaction steps in both samples, deceleration behavior characterized by concaved shapes was observed, possibly indicating that the process was controlled by diffusional removal of evolved water vapor.

### 2.3. Kinetic Deconvolution Analysis for the Thermal Decomposition of MB

**Figure 9:**
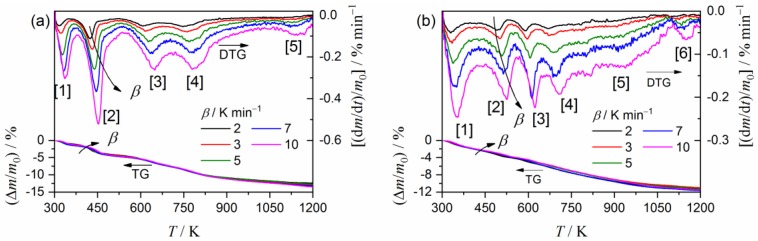
TG–DTG curves for the thermal decomposition of MB recorded at different *β* in a flow of air: (**a**) P-MB and (**b**) S-MB.

Figure 9 presents the TG–DTG curves recorded at different *β* values in a flow of air for the thermal decomposition of the synthesized P-MB and S-MB samples. The number of distinguishable DTG peaks was 5 and 6 for the thermal decomposition of P-MB and S-MB samples, respectively. In comparison with the TG–DTG curves for the clay mineral substrates (Figure 4), the third and fifth distinguishable DTG peaks in the thermal decomposition of P-MB appeared in addition to those expected from the thermal decomposition of palygorskite. For S-MB, the third and fourth distinguishable DTG peaks were the additional peaks. These additional peaks can be interpreted as the sublimation/decomposition of indigo incorporated into the clay mineral substrates.

**Figure 10:**
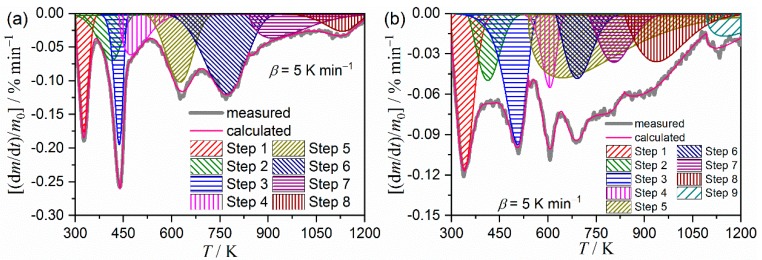
Typical results of MDA for the multistep thermal decomposition of MB samples: (**a**) P-MB and (**b**) S-MB.

MDA for the thermal decomposition of the MB samples was carried out by adding several minor peaks to the major discernable peaks in the DTG curves (Figure 9). Figure 10 illustrates typical results of MDA carried out by applying a Weibull function to each peak. By comparing the results of MDA for the thermal decomposition of clay mineral substrates, four additional peaks were revealed in both the P-MB and S-MB samples. These additional peaks (that is, the fourth, fifth, seventh, and eighth peaks for P-MB and the fourth, sixth, seventh, and eighth peaks for S-MB) are attributed to the thermal decomposition of the indigo molecules incorporated into the clay mineral matrix. The details of the analysis of the mathematically separated peaks are provided in Section S3 in the Supplementary Materials.

Further, the overall thermal decomposition of the MB samples was analyzed by KDA. The initial values of *c_i_* were substituted in for the values determined by MDA (Table S2). For the reaction steps attributed to the thermal decomposition of the clay mineral substrates, the initial values of the kinetic parameters were substituted with values from the results of the KDA for thermal decomposition of the substrates (Table 1). For the reaction steps attributed to the thermal decomposition of indigo, the apparent *E*_a,*i*_ values determined for the corresponding steps using MDA (Table S2) were used as the initial values. The *f_i_*(*α_i_*) for the reaction steps of the thermal decomposition of indigo were set to be SB(0, 1, 0) as the initial setting. Then, the order of apparent *A_i_* values was determined by graphically comparing the fit of the calculated curves with the experimental kinetic curve.

**Figure 11:**
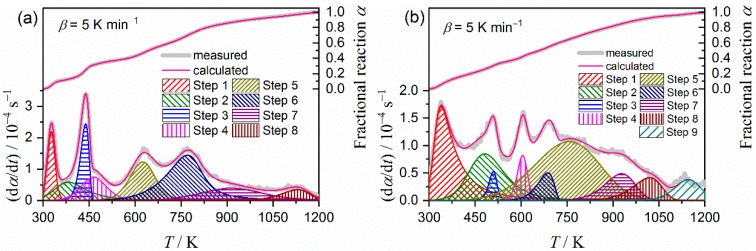
Typical results of KDA for the thermal decomposition of MB: (**a**) P-MB and (**b**) S-MB.

Figure 11 illustrates typical results of KDA for the thermal decomposition of the MB samples. The nearly perfect fit using Equation (1) with SB(*m*, *n*, *p*) in Equation (5) as the kinetic model function was realized by using the optimized kinetic parameters for each reaction step, as listed in Table 2. The optimized kinetic parameters for each reaction step from the overall kinetic curves recorded at different *β* values were practically invariant, as was determined by the acceptably small standard deviation values of each kinetic parameter. The sums of all the contributions from the reaction steps attributed to the thermal decomposition of indigo, that is, *i* = 4, 5, 7, and 8 for the P-MB sample and *i* = 4, 6, 7, and 8 for the S-MB sample, were 0.415 and 0.226, respectively. Compared to the initial sample mass *m*_0_, the fractional mass loss attributed to the thermal decomposition of indigo were calculated to be 5.35% and 2.58% for the P-MB and S-MB samples, respectively. These fractional mass-loss values are smaller than the initial mass ratio of indigo added to the clay mineral, 5.66%, before heat treatment and purification, in both samples. The difference in the mass-loss fractions between both samples indicates that the P-MB sample incorporates twice as much indigo as the S-MB sample.

**Figure 12:**
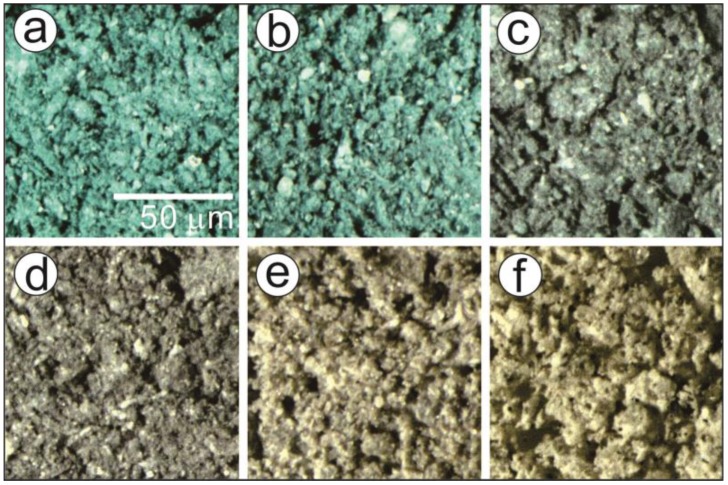
Typical optical microscopic views of the P-MB heated to different temperatures: (**a**) original sample, (**b**) 573 K, (**c**) 713 K, (**d**) 793 K, (**e**) 1003 K, and (**f**) 1223 K.

Figure 12 presents typical optical microscopic views of the P-MB sample as it was heated to different temperatures at *β* = 10 K min^−1^ in flowing air. The brilliant blue color of the original P-MB (Figure 12a) was maintained until the sample reached 573 K (Figure 12b), which is a higher temperature than the completion of the fourth reaction step, i.e., the first reaction step of the thermal decomposition of the indigo. This observation supports our previous assumption that the first decomposition step of indigo is the sublimation/decomposition of indigo adsorbed on the surface of the clay mineral substrate, not the indigo incorporated into the clay mineral matrix. Color degradation was observed in the temperature range that corresponds to the fifth reaction step, which is the second decomposition step of incorporated indigo (Figure 12b–d). Thus, the second decomposition step of indigo was interpreted as the decomposition of indigo incorporated into the micropores of the clay mineral substrate. The grayish color of the sample after the fifth reaction step was completed (Figure 12d) was due to the products of indigo decomposition. Upon further heating, the grayish color gradually disappeared after the seventh and eighth reaction steps (Figure 12e,f, respectively), which corresponded to the third and fourth decomposition steps of indigo, respectively. This observation was understood to be the oxidative decomposition of the residues in the flowing air atmosphere.

To compare the thermal decomposition behavior between the indigo incorporated into the MB and pure indigo crystals, thermally induced changes in pure indigo were subjected to a formal kinetic study. Approximately 97.8 ± 0.1% of mass loss was observed during the thermally induced sublimation/decomposition of the pure indigo crystals. The residue (2.2%) was lost by the oxidative decomposition at a higher temperature. The sublimation/decomposition process was characterized by a constant *E*_a_ value of 150.7 ± 4.7 kJ mol^−1^ and a phase-boundary controlled model. The details of the kinetic analysis are described in Section S4 in the Supplementary Materials.

**Figure 13:**
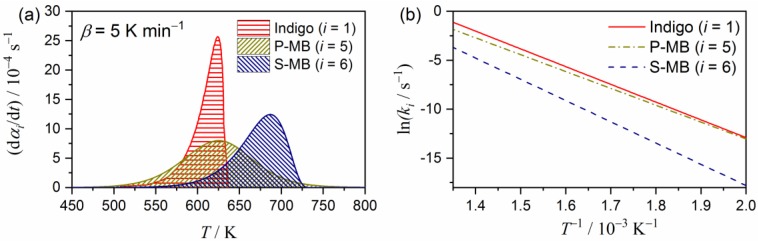
Comparison of the kinetic characteristics for the thermally induced sublimation/decomposition of indigo as crystalline particles, incorporated in P-MB (*i* = 5), and in S-MB (*i* = 6): (**a**) Kinetic curves and (**b**) Arrhenius plots.

Figure 13 compares the extracted kinetic curve (*β* = 5 K min^−1^) and Arrhenius plots for the respective reaction steps, drawn using the optimized Arrhenius parameters listed in Table 2 for the thermal decomposition of indigo incorporated in P-MB (*i* = 5) and S-MB (*i* = 6), with those for the thermally induced sublimation/decomposition of pure indigo crystals. Although the thermal decomposition of indigo in P-MB starts at roughly the same temperature, the reaction proceeds at a slower rate and continues to higher temperatures in comparison to the pure indigo crystals (Figure 13a). Despite the differences in the kinetic data, the Arrhenius plots for these reactions are comparable (Figure 13b), as follows: (*E*_a_/kJ mol^−1^, *A*/s^−1^) values for P-MB (*i* = 5) are (143.2 ± 0.3, (1.99 ± 0.02) × 10^9^). Mechanistic differences are a possible reason for the different kinetic behaviors. By contrast, the kinetic data and the Arrhenius plot for the thermal decomposition of the indigo incorporated into S-MB are very different from those of other indigo, having a higher thermal stability and a slower reaction rate. The larger Arrhenius parameters for S-MB (*i* = 6), i.e., (180.6 ± 0.7, (1.36 ± 0.01) × 10^11^), explain the difference between its thermal behavior and that of P-MB.

**Figure 14:**
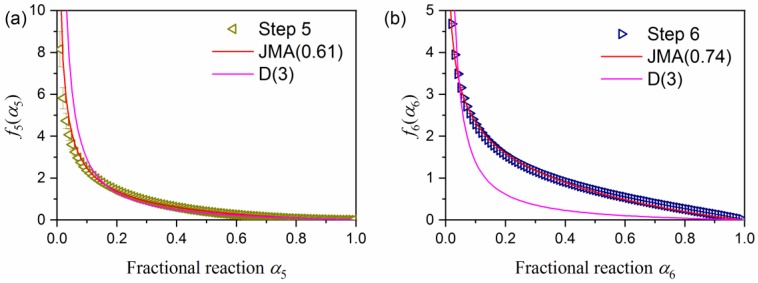
The plot of *f_i_*(*α_i_*) (= SB(*m_i_*, *n_i_*, *p_i_*)) versus *α_i_* for the thermal decomposition step of indigo incorporated into the pores of the MB samples: (**a**) P-MB (*i* = 5) and (**b**) S-MB (*i* = 6).

Figure 14 is the plot of *f_i_*(*α_i_*) (= SB(*m_i_*, *n_i_*, *p_i_*)) versus *α_i_* for the thermal decomposition step of indigo incorporated into the pores of the substrate minerals, i.e., P-MB (*i* = 5) and S-MB (*i* = 6). In both samples, the curves exhibit a concaved shape, which is characteristic of the deceleration process being controlled by diffusion. The curve was empirically fitted by a model for nucleation and growth controlled by diffusion, i.e., JMA(*m*) with *m* < 1 [55,56,57,58], or for three-dimensional shrinkage of reactant particle controlled by diffusion, i.e., the Jander model, D(3) [59].

(7) $$\mathrm{JMA}\left(m\right):\;f\left(\alpha \right)=m\left(1-\alpha \right){\left[-\ln\left(1-\alpha \right)\right]}^{1-1/m}$$

(8) $$D\left(3\right):\;f\left(\alpha \right)=\frac{3{\left(1-\alpha \right)}^{2/3}}{2\left[1-{\left(1-\alpha \right)}^{1/3}\right]}$$

The removal of gaseous products, which include sublimated indigo, by diffusion from the pores of the substrate mineral is likely the rate-limiting step. A reaction mechanism that is controlled by diffusion is very different from that of the sublimation/decomposition of pure indigo crystals (Figure S22c), which is also one reason for the thermal stability of the indigo incorporated into MB samples.

## 3. Materials and Methods

### 3.1. Sample Preparation

The fibrous clay minerals, i.e., palygorskite and sepiolite, were purchased from SEPIO Japan. Each clay mineral was ground using an agate mortar and a pestle. The ground sample was sieved to different particle sizes using a series of sieves with different mesh sizes and an electric shaking apparatus (MVS-1, AS ONE). The sample particles sieved to 170–200 μm in diameter were used to synthesize MB. The clay mineral samples were immersed in 1 M-HCl(aq) and the slurry was stirred for approximately 10 h to remove the CaCO_3_ impurity included in the clay mineral [60]. The precipitate was filtered and washed repeatedly until the chloride ions were not detected in the filtrates against AgNO_3_(aq). The separated clay minerals were dried in an electric oven (DK240S, YAMATO) at 343 K for 24 h.

MB was prepared following previously reported procedures [31,33,35,40,41,43,61]. The clay minerals treated with HCl(aq) were used as the substrates for synthesizing MB. Indigo (≥95.0%, NACALAI Tesque) was used as the pigment. Indigo and the clay mineral substrate were mixed in the following mass ratios: 0.01, 0.02, 0.06, or 0.10. Approximately 2.5 g of the mixed sample was placed into a ceramic crucible and covered with a ceramic lid. Samples were heated in an electric furnace (KDF P-70, DENKEN) at 368 K for 24 h and subsequently at 413 K for 24 h.

Excess indigo that was not reacted with the clay mineral substrate was removed as follows [62]. Approximately 0.5 g of the heat-treated sample was dispersed into 20 mL of a 0.25 M-NaOH(aq) solution, to which 0.5 g of sodium dithionate (Na_2_S_2_O_4_) had been dissolved. The samples were kept at 348 K and stirred using a magnetic stirrer for 5 min. After filtration, the separated solid was washed repeatedly with water and dried in air.

### 3.2. Sample Characterization

The clay mineral samples were identified using powder X-ray diffractometry (XRD) and FT-IR. Samples were press-fitted to sample holders to carry out XRD measurement on a diffractometer (RINT-2200V, Rigaku, Tokyo, Japan) with a radiation source (Cu-Kα, 40 kV, 20 mA) in the 2*θ* range of 5–60° at a scan speed of 4° min^−1^. FT-IR spectra were measured in a wavenumber range of 400–4600 cm^−1^ using the diffuse reflectance method in a spectrophotometer (FT-IR8400S, Shimadzu, Kyoto, Japan) after diluting the sample with KBr. The clay mineral substrates and MB samples were subjected to simultaneous TG–DTA, UV-Vis spectroscopy, and morphological observation using a SEM. Approximately 10 mg of each sample was weighed into a platinum pan (5 mm in diameter and 2.5 mm in height) and heated at a *β* of 10 K min^−1^ from room temperature to 1223 K in flowing N_2_ (flowrate: 300 cm^3^ min^−1^) for recording TG–DTA curves using an instrument (STA7300, Hitachi High-Tech. Sci., Tokyo, Japan). The UV-Vis spectra of the samples press-fitted to a glass slide were recorded in a wavelength range of 400–700 nm using a spectrophotometer (V-560, JASCO, Tokyo, Japan) equipped with an integrating sphere. For SEM observations, the sample was coated with a thin Pt layer by sputtering (30 mA, 40 s, JFC-1600, JEOL, Tokyo, Japan) and observed using an instrument (JSM-6510, JEOL).

### 3.3. Tracking of the Thermal Decomposition Process

Thermal behaviors of the clay minerals received and those that were treated with HCl(aq) were investigated by TG/DTA–mass spectrometry (MS). Approximately 10 mg of each sample was weighed into a platinum pan (5 mm in diameter and 2.5 mm in height) and TG–DTA measurements were carried out using an instrument (Thermoplus TG-8120, Rigaku). The sample was heated from room temperature to 1223 K in flowing He (200 cm^3^ min^−1^). During the TG–DTA measurement, the outlet gas from the instrument was transferred into a MS instrument (M-200QA, Anelva, Kanagawa, Japan) through a capillary tube (0.007 mm in inner diameter and 0.8 m in length) that was heated at 500 K. MS measurements (EMSN: 1.0 A; SEM: 1.0 kV) for the outlet gas were continuously repeated in a *m*/*z* range of 10–50.

Using the TG–DTA instrument (STA7300), approximately 10 mg of MB samples were heated to different temperatures at *β* = 10 K min^−1^ in flowing air (300 cm^3^ min^−1^). After the sample was cooled to room temperature, the partially decomposed samples were observed using an optical microscope (SZX7, Olympus, Tokyo, Japan) for recording the color of the sample.

### 3.4. Measurement of the Kinetic Data for the Thermal Decomposition

To record the kinetic data for the thermal decomposition of the clay mineral substrates treated with HCl(aq) and the MB samples, TG–DTA measurements were carried out using the STA7300 instrument. Approximately 5 mg of each sample was weighed into a platinum pan (5 mm in diameter and 2.5 mm in height) and heated from room temperature to 1223 K at various *β* values between 2 and 10 K min^−1^ in flowing N_2_ or air (flowrate: 300 cm^3^ min^−1^). The TG–DTA instrument was previously calibrated in view of mass change values and temperature using standard procedures.

## 4. Conclusions

Thermal decompositions of palygorskite and sepiolite were characterized by three and four distinguished DTG peaks, respectively, which were attributed to the dehydration of zeolite, coordinating, and structural water. Kinetically, the second mass-loss process in both palygorskite and sepiolite was further separated into two reaction steps. Consequently, the thermal decompositions of palygorskite and sepiolite were kinetically separated into four and five reaction steps, respectively. The processes occurring in MB samples, i.e., P-MB and S-MB, were separated by KDA into eight and nine reaction steps, respectively. The additional four reaction steps that were observed for MB samples are attributed to the thermal decomposition of the indigo incorporated into the clay mineral substrates. Discoloration of MB samples occurred during the second reaction step of the thermal decomposition of the indigo incorporated into the clay mineral substrates. The second reaction step is expected to be directly correlated to the thermal stability of the MB samples as a pigment. The second step of the thermal decomposition of indigo in P-MB started at approximately the same temperature as the thermally induced sublimation/decomposition of pure indigo crystals. In addition, the apparent Arrhenius parameters evaluated for these reaction processes were also comparable. However, the second step in the thermal decomposition of indigo in P-MB occurs at a slower rate and continues to higher temperatures compared to pure indigo crystals. This is explained by different physico-geometrical reaction mechanisms; the thermally induced sublimation/decomposition of pure indigo crystals is a phase boundary-controlled reaction and the second reaction step of indigo decomposition in P-MB is a diffusion-controlled reaction. Even though the second reaction step of indigo decomposition in S-MB started at a higher temperature in comparison to that in P-MB, both reactions ended at around the same temperature. The difference in starting temperatures can be explained by the larger Arrhenius parameters for S-MB. Although the stability of MB is commonly discussed in connection with the strength of the chemical bonds between indigo and the clay mineral substrate, the present study indicates that the physico-geometrical kinetic behavior of the thermal decomposition of indigo incorporated into the clay mineral substrate is another important factor in discussing the thermal stability of MB.

## Figures and Tables

**Table 1:** The average kinetic parameters optimized by KDA for each reaction step of the thermal decomposition of the purified clay minerals.

Sample	*i*	*c_i_*	*E*_a,*i*_/kJ mol^−1^	*A_i_*/s^−1^	*f_i_*(*α_i_*)=*α_i_^m^*(1−*α_i_*)*^n^*[−ln(1−*α_i_*)]*^p^*			*R^2^*
					*m*	*n*	*p*	
Palygorskite	1	0.32 ± 0.01	63.7 ± 0.2	(4.35 ± 0.07) × 10^7^	−0.63 ± 0.04	1.26 ± 0.03	0.73 ± 0.03	0.99 ± 0.01
	2	0.12 ± 0.02	90.5 ± 1.7	(2.94 ± 0.01) × 10^8^	−0.33 ± 0.02	1.13 ± 0.09	–0.35 ± 0.02	
	3	0.11 ± 0.01	114.4 ± 0.2	(2.00 ± 0.02) × 10^11^	0.03 ± 0.01	0.61 ± 0.07	0.21 ± 0.01	
	4	0.45 ± 0.01	190.0 ± 1.5	(4.79 ± 0.05) × 10^8^	–32.8 ± 3.8	13.8 ± 1.7	29.5 ± 3.7	
Sepiolite	1	0.31 ± 0.01	80.4 ± 0.1	(3.25 ± 0.02) × 10^10^	0.33 ± 0.02	2.34 ± 0.04	–0.27 ± 0.01	0.99 ± 0.01
	2	0.14 ± 0.01	64.6 ± 0.9	(1.28 ± 0.01) × 10^4^	−0.01 ± 0.01	2.30 ± 0.05	–0.59 ± 0.01	
	3	0.04 ± 0.01	213.0 ± 1.0	(1.01 ± 0.01) × 10^19^	–0.02 ± 0.01	1.41 ± 0.02	–0.09 ± 0.01	
	4	0.41 ± 0.01	124.3 ± 1.4	(2.37 ± 0.01) × 10^5^	–0.46 ± 0.01	2.93 ± 0.05	–1.43 ± 0.02	
	5	0.10 ± 0.01	738.4 ± 1.6	(9.50 ± 0.01) × 10^32^	–1.29 ± 0.01	2.30 ± 0.01	–1.78 ± 0.02	

**Table 2:** Kinetic parameters for each reaction step for the thermal decomposition of MB, as determined by KDA.

Sample	*i*	*c_i_*	*E*_a,*i*_*/*kJ mol^−1^	*A_i_/*s^−1^	*f_i_*(*α_i_*)=*α_i_^m^*(1 − *α_i_*)*^n^*[−ln(1 − *α_i_*)]*^p^*			*R* ^2^
					*m*	*n*	*p*	
P-MB	1	0.08 ± 0.01	61.7 ± 0.5	(4.33 ± 0.02) × 10^7^	–0.57 ± 0.10	1.69 ± 0.09	0.73 ± 0.05	0.99 ± 0.01
	2	0.10 ± 0.01	46.4 ± 0.3	(1.95 ± 0.01) × 10^3^	−0.04 ± 0.01	2.68 ± 0.22	–0.18 ± 0.01	
	3	0.10 ± 0.01	113.0 ± 0.6	(1.70 ± 0.01) × 10^11^	0.02 ± 0.01	0.85 ± 0.01	–0.07 ± 0.01	
	4	0.09 ± 0.01	43.7 ± 2.0	(1.96 ± 0.02) × 10^2^	1.19 ± 0.06	1.05 ± 0.03	–1.10 ± 0.05	
	5	0.15 ± 0.03	143.2 ± 0.3	(1.99 ± 0.02) × 10^9^	–0.11 ± 0.01	1.64 ± 0.17	–0.35 ± 0.03	
	6	0.31 ± 0.03	183.2 ± 2.5	(4.99 ± 0.03) × 10^8^	–26.61 ± 3.69	11.94 ± 2.27	24.50 ± 4.38	
	7	0.13 ± 0.01	634.3 ± 3.6	(1.70 ± 0.01) × 10^32^	–2.44 ± 0.19	3.79 ± 0.57	–3.82 ± 0.43	
	8	0.05 ± 0.01	448.3 ± 2.8	(8.84 ± 0.08) × 10^17^	–0.39 ± 0.03	1.21 ± 0.12	–0.48 ± 0.06	
S-MB	1	0.22 ± 0.01	82.4 ± 0.5	(2.16 ± 0.01) × 10^10^	0.25 ± 0.01	5.57 ± 0.09	–0.35 ± 0.01	0.98 ± 0.02
	2	0.15 ± 0.01	64.5 ± 0.8	(1.29 ± 0.01) × 10^4^	−0.01 ± 0.01	2.45 ± 0.08	–0.40 ± 0.01	
	3	0.02 ± 0.01	206.2 ± 1.9	(9.68 ± 0.01) × 10^18^	–0.02 ± 0.01	1.26 ± 0.01	–0.11 ± 0.01	
	4	0.05 ± 0.01	160.3 ± 0.4	(4.48 ± 0.02) × 10^11^	0.06 ± 0.01	1.35 ± 0.08	0.24 ± 0.01	
	5	0.34 ± 0.01	127.9 ± 2.4	(2.33 ± 6.98) × 10^5^	–0.46 ± 0.01	1.17 ± 0.02	–1.39 ± 0.05	
	6	0.04 ± 0.01	180.6 ± 0.7	(1.36 ± 0.01) × 10^11^	–0.20 ± 0.01	0.78 ± 0.01	–0.20 ± 0.01	
	7	0.08 ± 0.01	120.8 ± 3.3	(9.94 ± 0.02) × 10^3^	0.06 ± 0.01	1.07 ± 0.01	0.12 ± 0.01	
	8	0.06 ± 0.01	314.0 ± 5.5	(1.51 ± 0.01) × 10^13^	–0.40 ± 0.01	1.09 ± 0.01	–0.33 ± 0.01	
	9	0.05 ± 0.01	780.9 ± 9.1	(9.65 ± 0.01) × 10^32^	–0.73 ± 0.01	2.14 ± 0.03	–0.66 ± 0.01	

## Supplementary Materials

The following are available online, S1: Sample preparation and characterization (Figures S1–S13), S2: Kinetic analysis for the thermal decomposition of clay mineral substrate (Table S1, Figures S14–S16), S3: Kinetic analysis for the thermal decomposition of Maya blue (Table S2, Figures S17–S20), S4: Kinetic analysis for the thermally induced sublimation/decomposition of indigo (Figures S21 and S22).
