# Supplementary material for: Thermal Decomposition of Maya Blue: Extraction of Indigo Thermal Decomposition Steps from a Multistep Heterogeneous Reaction Using a Kinetic Deconvolution Analysis
Source: Molecules. 2019 Jul 9;24(13):2515. doi: 10.3390/molecules24132515 (PMC6651835; doi:10.3390/molecules24132515)
Supplement: Supplementary file 1 [file molecules-24-02515-s001.pdf]

molecules

Supplementary Material

Thermal Decomposition of Maya Blue: Extraction of Indigo Thermal Decomposition Steps from a Multistep Heterogeneous Reaction Using a Kinetic Deconvolution Analysis

Yui Yamamoto and Nobuyoshi Koga*

Chemistry Laboratory, Department of Science Education, Graduate School of Education, Hiroshima University, 1-1-1 Kagamiyama, Higashi-Hiroshima 739-8524, Japan

Contents

S1. Sample preparation and characterization S3

(1) Clay mineral substrates S3

Figure S1. Comparisons of the XRD patterns of the purchased clay minerals and those treated with HCl(aq): (a) palygorskite and (b) sepiolite. S3

Figure S2. Comparison of the FT-IR spectra of the purchased clay minerals before and after treatment with HCl(aq): (a) palygorskite and (b) sepiolite. S4

Figure S3. Comparison of the TG–DTG–DTA curves of the purchased clay minerals before and after treatment with HCl(aq): (a) palygorskite and (b) sepiolite. S4

Figure S4. TG–DTA curves and mass thermograms for *m*/*z* = 18 (H_2O^+) and *m*/*z* = 44 (CO_2^+) for the palygorskite sample before and after treatment with HCl(aq). S4

Figure S5. TG–DTA curves and mass thermograms for *m*/*z* = 18 (H_2O^+) and *m*/*z* = 44 (CO_2^+) for the sepiolite sample before and after treatment with HCl(aq). S4

(2) Preparation of Maya blue S5

Figure S6. Coloration comparisons of the indigo–palygorskite mixtures with different ratios before and after heating. S5

Figure S7. Coloration comparisons of the indigo–sepiolite mixtures with different ratios before and after heating. . S5

Figure S8. UV-Vis spectra of the starting materials and the heat-treated samples: (a) starting materials, (b) heat treated palygorskite–indigo mixtures, and (c) heat treated sepiolite–indigo mixtures. S5

Figure S9. TG–DTG–DTA curves for pure indigo crystals. S6

Figure S10. Comparison of the TG–DTG–DTA curves for the heat-treated indigo/sepiolite samples before and after $Na_2S_2O_4$(aq) the treatment recorded in (a) flowing N_2 and (b) flowing air. S6

Figure S11. Coloration comparisons of the of the heat-treated indigo/clay mineral samples before and after treatment with $Na_2S_2O_4$ (aq): (a) indigo/palygorskite and (b) indigo/sepiolite. S6

Figure S12. Comparison of the UV-Vis spectra of the heat-treated indigo/clay mineral samples before and after treatment with $Na_2S_2O_4$ (aq): (a) indigo/palygorskite and (b) indigo/sepiolite. S6

Figure S13. Typical SEM images of the synthesized MB: (a) P-MB and (b) S-MB. S6

S2. Kinetic analysis for the thermal decomposition of the clay mineral substrate S7

* Corresponding author, e-mail: nkoga@hiroshima-u.ac.jp

Weibull function: S7

Table S1. Contribution c_i of each reaction step *i* to the total mass-loss value for the overall thermal decomposition of the clay mineral substrates as determined by MDA, and $E_{a,i}$ values determined by the Friedman plot applied to the kinetic curves for each individual reaction step separated by MDA S7

Figure S14. Kinetic curves for each reaction step of the multistep thermal decomposition of palygorskite, generated by MDA of the overall kinetic curves: (a) first reaction step, (b) second reaction step, (c) third reaction step, and (d) fifth reaction step. S8

Figure S15. Kinetic curves for each reaction step of the multistep thermal decomposition of sepiolite, generated by MDA of the overall kinetic curves: (a) first reaction step, (b) second reaction step, (c) third reaction step, (d) fourth reaction step, and (e) fifth reaction step. S8

Figure S16. E_a values at different α_i for each reaction step *i* of the multistep thermal decomposition for the clay mineral substrates: (a) palygorskite and (b) sepiolite. S9

S3. Kinetic analysis for the thermal decomposition of Maya blue S9

Table S2. Contributions c_i of each reaction step *i* to the total mass-loss value for the overall thermal decomposition of the MB samples as determined by MDA and $E_{a,i}$ values, which have been determined by the Friedman plot applied to the kinetic curves for each individual reaction step separated by MDA S9

Figure S17. Kinetic curves for each reaction step of the multistep thermal decomposition of the P-MB sample, generated by MDA: (a) first, (b) second, (c) third, (d) fourth, (e) fifth, (f) sixth, (g) seventh, and (h) eighth reaction steps. S10

Figure S18. Kinetic curves for each reaction step of the multistep thermal decomposition of the S-MB sample, generated by MDA: (a) first, (b) second, (c) third, (d) fourth, (e) fifth, (f) sixth, (g) seventh, (h) eighth, and (i) ninth reaction steps. S11

Figure S19. Changes in $E_{a,i}$ with α_i, as determined by the Friedman plot applied to the kinetic curves generated by MDA for the reaction steps attributed to the thermal decomposition of indigo in an MB sample: (a) P-MB and (b) S-MB samples. S12

Figure S20. Typical optical microscopic views of S-MB when heated to different temperatures: (a) original sample, (b) 573 K, (c) 653 K, (d) 753 K, (e) 1003 K, and (f) 1093 K. S12

S4. Kinetic analysis for the thermally induced sublimation/decomposition of indigo S12

Figure S21. A series of TG–DTG–DTA curves for the indigo reagent (m_0 = 1.0 mg) recorded at different β values in flowing air (300 cm^3 min^{-1}). S13

Figure S22. Typical result of the isoconversional kinetic analysis for the first mass-loss process of sublimation/decomposition of indigo reagent: (a) Friedman plots at different α values, (b) E_a value at different α values, and (c) experimental master plot of $(d\alpha_1/d\theta_1)$ versus α_1. .... S13

References S13

S1. Sample preparation and characterization

(1) Clay mineral substrates

Figure S1 compares XRD patterns of the purchased clay minerals and those that were treated with HCl(aq). The purchased samples, both palygorskite and sepiolite, exhibited XRD patters composed of clay mineral and calcite. The XRD peaks attributed to calcite disappear completely after the samples are treated with HCl(aq), whereas the clay mineral peaks are maintained. The XRD patterns of the purified clay minerals match the reported XRD patterns of palygorskite ($Mg_{2.074}Al_{1.026}(Si_4O_{10.48})_2(OH)_2(H_2O)_{10.68}$, orthorhombic, S.G. = Pbmn(53), a = 12.7630, b = 17.8420, c = 5.2410, ICCD PDF 01-082-1873) [S1, S2] and sepiolite ($Mg_8(OH)_4Si_{12}O_{30}(H_2O)_{12}$, orthorhombic, S.G. = Pncn(52), a = 13.4000, b = 26.8000, c = 5.2800, ICCD PDF 01-075-1597) [S3], respectively. Removal of the calcite phase by treatment with HCl(aq) was also confirmed by comparing the FT-IR spectra of the purchased clay minerals and those treated with HCl(aq), as illustrated in Figure S2. The purchased samples exhibited absorptions attributed to the ν_2 and ν_3 modes of CO_3^{2-} vibration at 881 cm^{-1} and in the range of 1430–1500 cm^{-1}, respectively [S4, S5], in addition to an Si–O stretching band in the range of 914–1202 cm^{-1}, O–H bending mode at 1663 cm^{-1}, and H–O–H stretching band in the range of 3000–4000 cm^{-1} attributed to the clay minerals [S6-S8]. All the absorption peaks that originated from CO_3^{2-} disappear after treatment with HCl(aq). Figure S3 compares the TG–DTG–DTA curves for the purchased samples and those treated with HCl(aq). The thermal decomposition step of calcite observed for the purchased sample in the temperature range of approximately 825–1000 K is not detected in the samples treated with HCl(aq). It should be noted that the mass-loss behavior of the other reaction steps did not change after treatment with HCl(aq), except a slight change in the mass-loss behavior of the sepiolite sample near room temperature, which is attributed to the dehydration of zeolite water. From the mass-loss value during the thermal decomposition of calcite in the purchased samples, the content of calcite was determined to be 21.6% and 15.4% for the palygorskite and sepiolite samples, respectively. The disappearance of the thermal decomposition step of calcite after treatment with HCl(aq) was further evidenced by the TG/DTA–MS measurements, as illustrated in Figures S4 and S5 for the palygorskite and sepiolite samples, respectively. In both samples, the purchased mineral exhibits the evolution steps of H_2O and CO_2. However, only H_2O evolution steps are observed for samples treated with HCl(aq). The H_2O evolution behavior does not practically change after the HCl(aq) treatment in both the clay mineral samples, especially for the palygorskite sample.

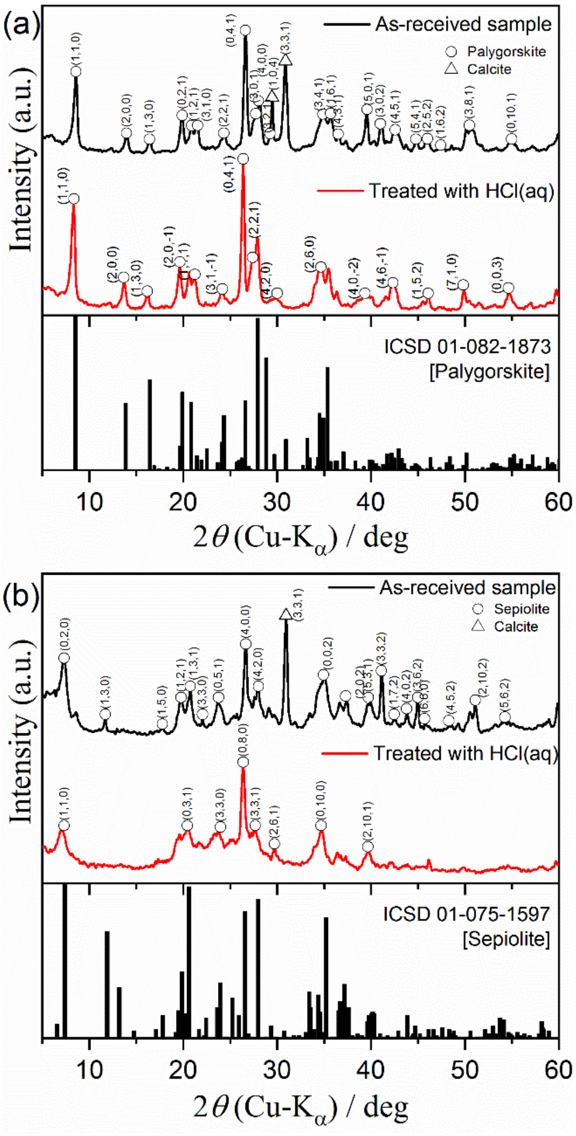

Figure S1. Comparisons of the XRD patterns of the purchased clay minerals and those treated with HCl(aq): (a) palygorskite and (b) sepiolite.

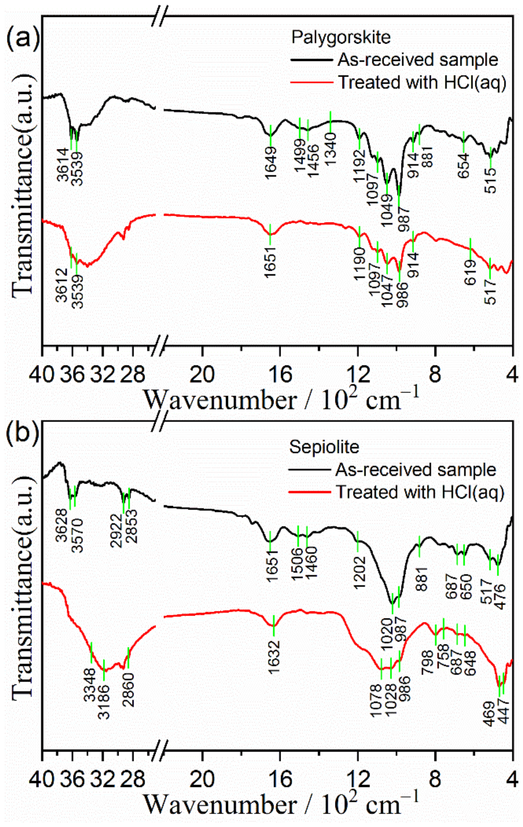

Figure S2. Comparison of the FT-IR spectra of the purchased clay minerals before and after treatment with HCl(aq): (a) palygorskite and (b) sepiolite.

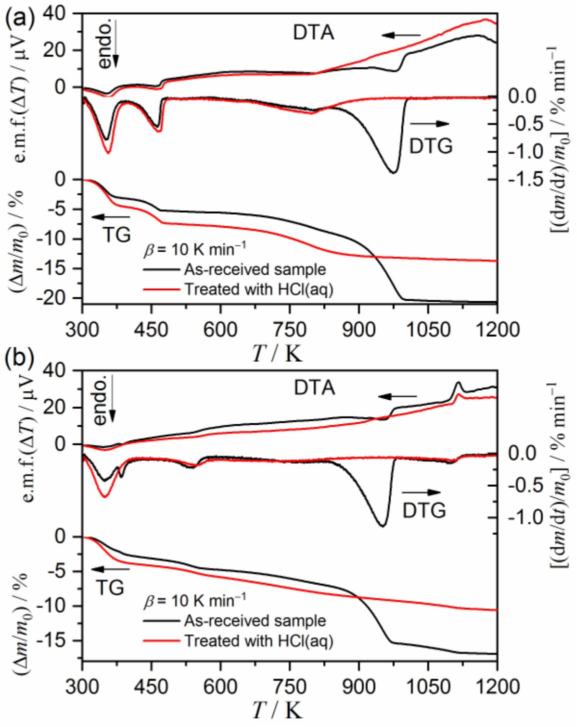

Figure S3. Comparison of the TG–DTG–DTA curves of the purchased clay minerals before and after treatment with HCl(aq): (a) palygorskite and (b) sepiolite.

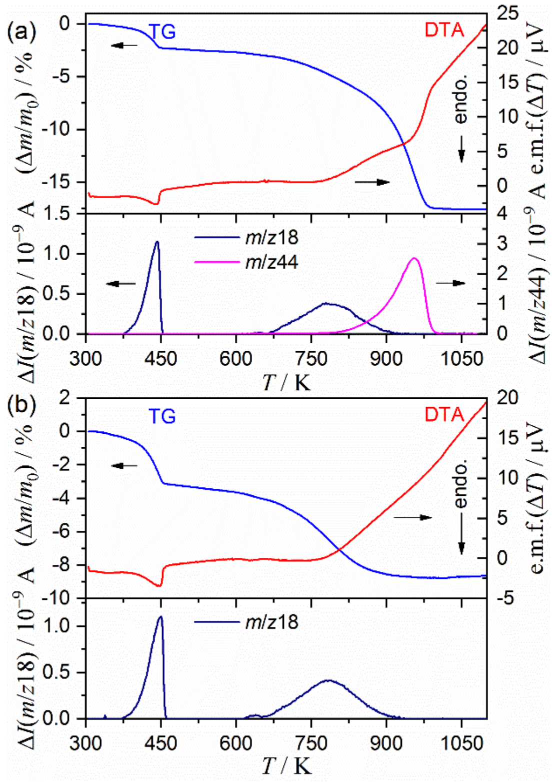

Figure S4. TG–DTA curves and mass thermograms for m/z = 18 (H_2O^+) and m/z = 44 (CO_2^+) for the palygorskite sample before and after treatment with HCl(aq).

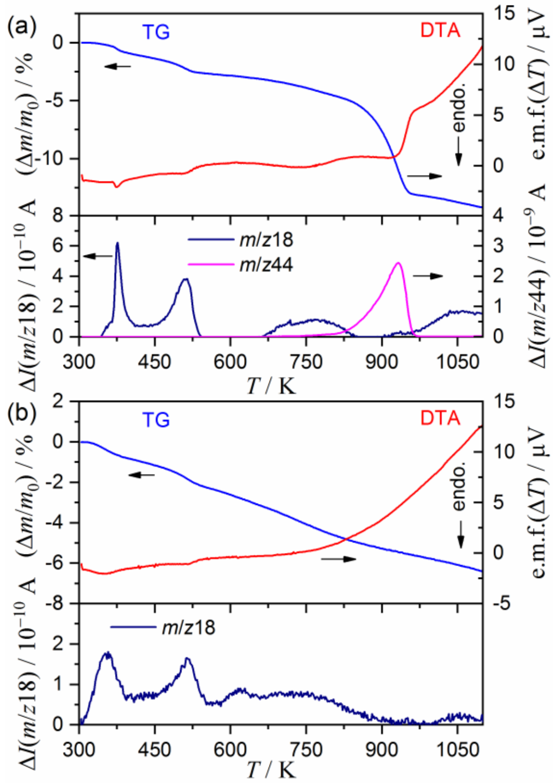

Figure S5. TG–DTA curves and mass thermograms for m/z = 18 (H_2O^+) and m/z = 44 (CO_2^+) for the sepiolite sample before and after treatment with HCl(aq).

(2) Preparation of Maya blue

Figures S6 and S7 compare the colorations of the indigo-clay mineral mixtures before and after heating the sample mixtures based on palygorskite and sepiolite, respectively. Regardless of the indigo/clay mineral ratio and the clay mineral substrate used, the blue color becomes clearer after heat treatment. The UV-Vis spectra of the heat-treated samples are presented in Figure S8, together with those for the starting materials, i.e., indigo, palygorskite, and sepiolite. The heat-treated samples have a broad absorption in the wavelength range of 550–700 nm, with a maximum absorption at approximately 650 nm, which is different from the absorption spectrum of indigo. As the ratio of indigo increases in the samples, an absorbance in the range of 425–600 nm increases, and the wavelength at the maximum absorbance gradually shifts to shorter wavelength, which occurs in addition to the increase in the absorbance in the overall range. The absorbances in the range of 550–700 nm for the sample with the indigo/clay mineral ratio of 0.06 to 0.10 are practically the same regardless of the clay mineral substrate. Based on these data, it is likely that the indigo–clay mineral interaction is saturated for the sample when the indigo/clay mineral ratio is larger than 0.06.

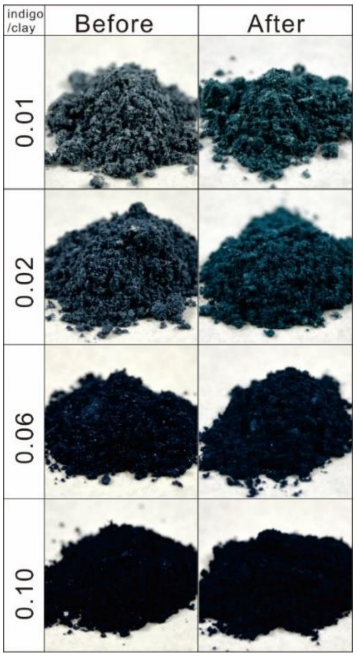

Figure S6. Coloration comparisons of the indigo–palygorskite mixtures with different ratios before and after heating.

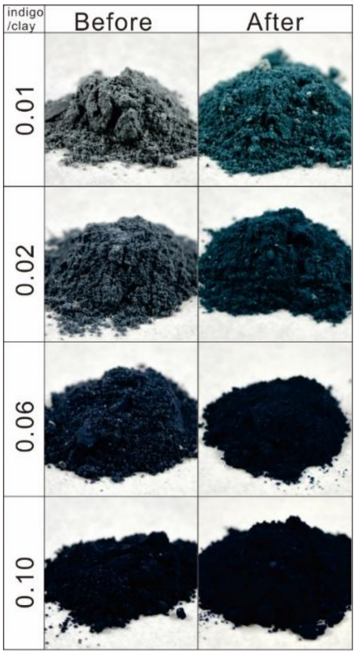

Figure S7. Coloration comparisons of the indigo–sepiolite mixtures with different ratios before and after heating.

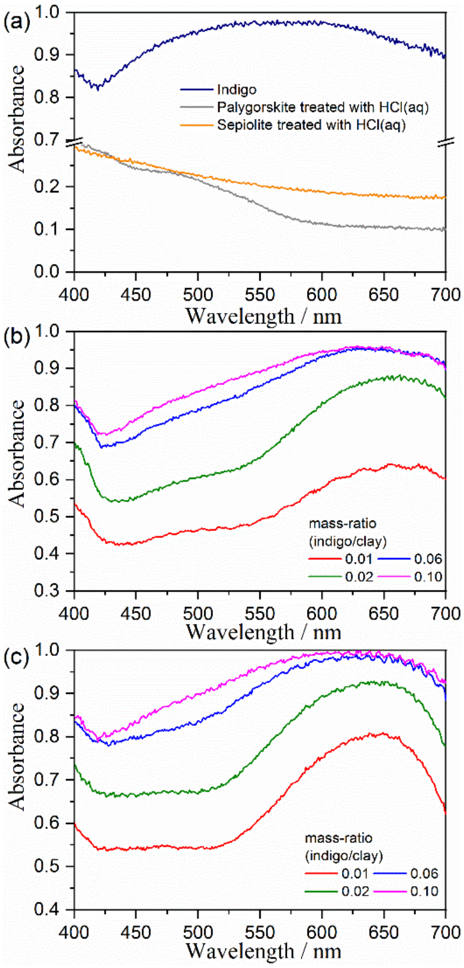

Figure S8. UV-Vis spectra of the starting materials and the heat-treated samples: (a) starting materials, (b) heat treated palygorskite–indigo mixtures, and (c) heat treated sepiolite–indigo mixtures.

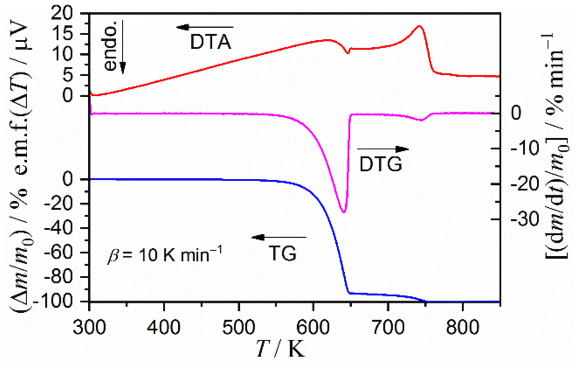

Figure S9. TG–DTG–DTA curves for pure indigo crystals.

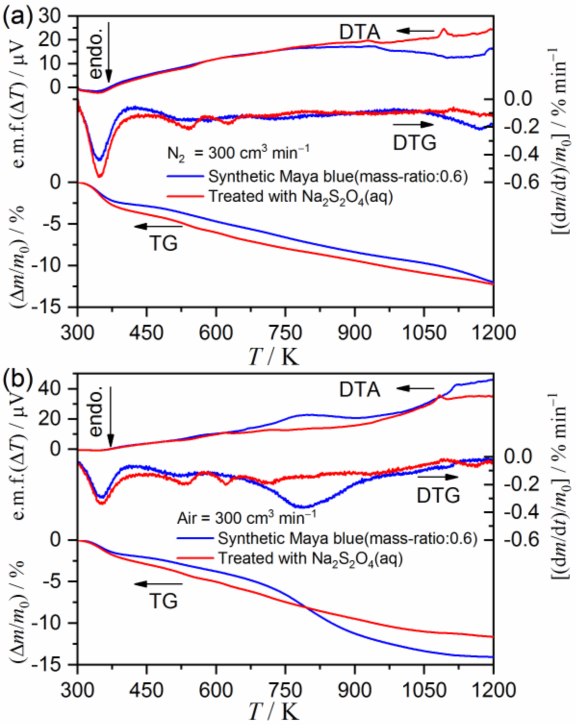

Figure S10. Comparison of the TG–DTG–DTA curves for the heat-treated indigo/sepiolite samples before and after $Na_2S_2O_4$(aq) the treatment recorded in (a) flowing N_2 and (b) flowing air.

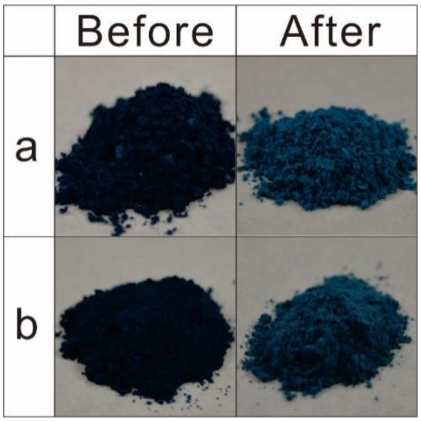

Figure S11. Coloration comparisons of the of the heat-treated indigo/clay mineral samples before and after treatment with $Na_2S_2O_4$(aq): (a) indigo/palygorskite and (b) indigo/sepiolite.

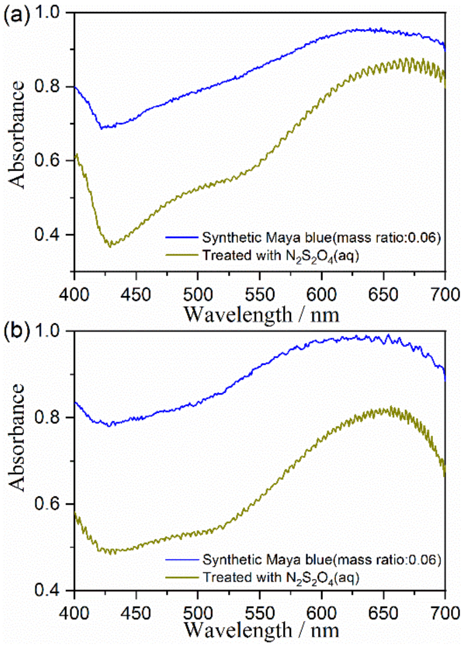

Figure S12. Comparison of the UV-Vis spectra of the heat-treated indigo/clay mineral samples before and after treatment with $Na_2S_2O_4$(aq): (a) indigo/palygorskite and (b) indigo/sepiolite.

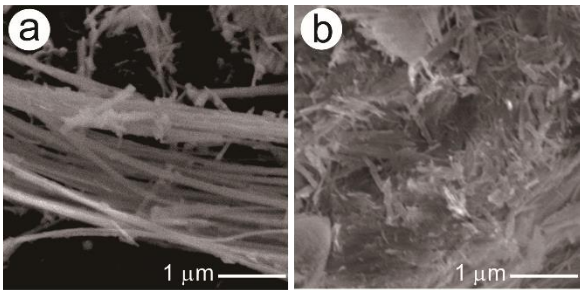

Figure S13. Typical SEM images of the synthesized MB: (a) P-MB and (b) S-MB.

S2. Kinetic analysis for the thermal decomposition of the clay mineral substrate

Weibull function:

$$F_i(t) = a_0\left(\frac{a_3-1}{a_3}\right)^{\frac{1-a_3}{a_3}}\left\{\frac{t-a_1}{a_2}+\left(\frac{a_3-1}{a_3}\right)^{\frac{1}{a_3}}\right\}^{a_3-1}\exp\left[-\left\{\frac{t-a_1}{a_2}+\left(\frac{a_3-1}{a_3}\right)^{\frac{1}{a_3}}\right\}^{a_3}+\frac{a_3-1}{a_3}\right] \quad \text{(S1)}$$

where a_0, a_1, a_2, and a_3 are the amplitude, center, width, and shape, respectively.

Figures S14 and S15 present the kinetic curves at different β values for each reaction step extracted from the overall kinetic curves by MDA for the thermal decomposition of palygorskite and sepiolite, respectively. In a series of kinetic curves for each reaction step, the curves shift systematically to higher temperatures with increasing β and can be subjected to the kinetic analysis by assuming a single-step reaction. The isoconversional kinetic analysis based on Eq. (3) was applied to all the series of the kinetic curves for each reaction step. Figure S16 illustrates the variation of E_a values at different α_i obtained as results of the Friedman plot. For several separated reaction steps, ideal results exhibiting constant $E_{a,i}$ values over a wide range of α_i were observed, for example, in the first, third, and fourth separated steps for the thermal decomposition of palygorskite and in the first and fourth steps for the thermal decomposition of sepiolite. The average value of $E_{a,i}$ within the α_i range with a satisfactory constant value or the systematic variation trend of $E_{a,i}$ are also listed in Table S1.

Table S1. Contribution c_i of each reaction step i to the total mass-loss value for the overall thermal decomposition of the clay mineral substrates as determined by MDA, and $E_{a,i}$ values determined by the Friedman plot applied to the kinetic curves for each individual reaction step separated by MDA

sample	step i	c_i	$E_{a,i}$ / kJ mol$^{-1,a}$
Palygorskite	1	33.1 ± 1.0	69.2 ± 3.6 (0.2 ≤ α_1 ≤ 0.8)
	2	11.5 ± 0.8	89.3 ± 21.3 (0.1 ≤ α_2 ≤ 0.8)
	3	12.3 ± 1.6	114.2 ± 1.6 (0.1 ≤ α_3 ≤ 0.9)
	4	43.2 ± 0.4	186.9 ± 1.7 (0.1 ≤ α_4 ≤ 0.9)
Spiolite	1	28.9 ± 0.8	81.9 ± 1.6 (0.2 ≤ α_1 ≤ 0.8)
	2	9.2 ± 0.8	63.4 ± 13.1 (0.2 ≤ α_2 ≤ 0.9)
	3	8.9 ± 1.1	214.4 ± 24.6 (0.2 ≤ α_3 ≤ 0.8)
	4	46.8 ± 2.2	112.7 ± 6.4 (0.3 ≤ α_4 ≤ 0.8)
	5	6.1 ± 1.5	741.5 ± 160.5 (0.2 ≤ α_5 ≤ 0.8)

[a] Averaged over the α_i range indicated in the blanket.

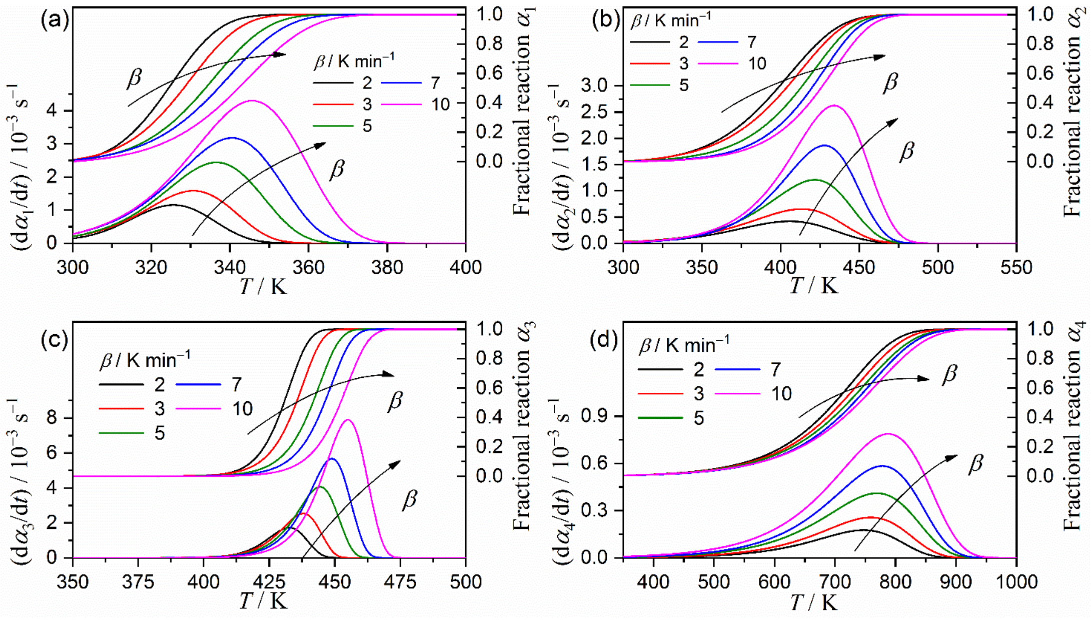

Figure S14. Kinetic curves for each reaction step of the multistep thermal decomposition of palygorskite, generated by MDA of the overall kinetic curves: (a) first reaction step, (b) second reaction step, (c) third reaction step, and (d) fifth reaction step.

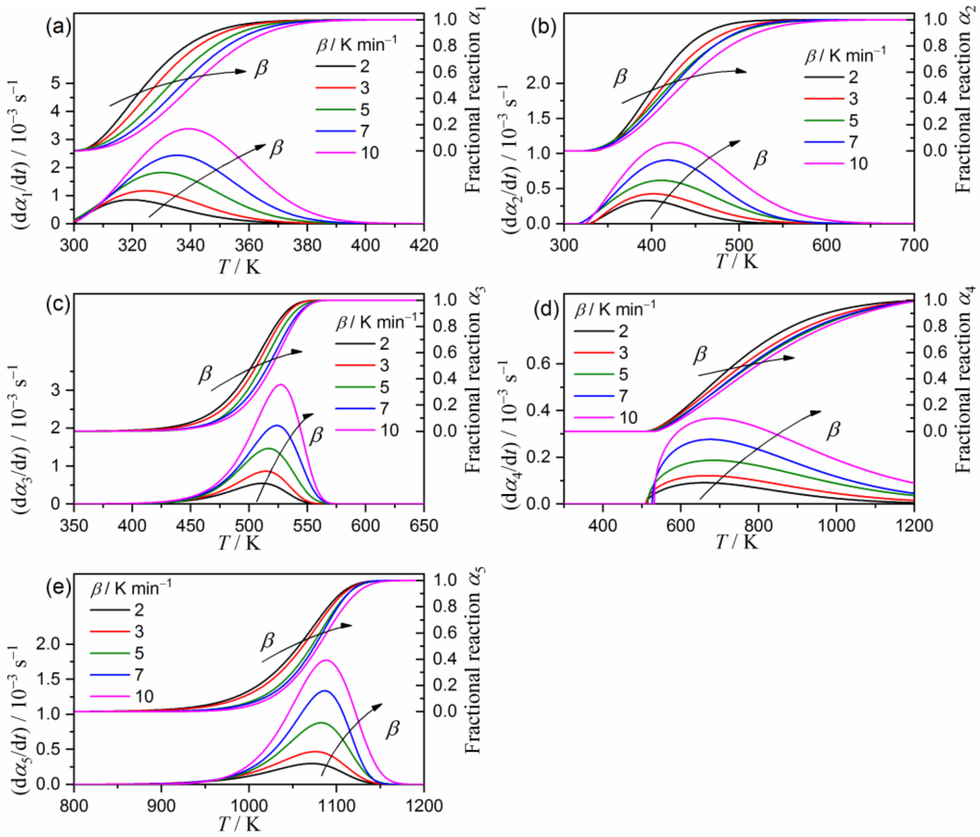

Figure S15. Kinetic curves for each reaction step of the multistep thermal decomposition of sepiolite, generated by MDA of the overall kinetic curves: (a) first reaction step, (b) second reaction step, (c) third reaction step, (d) fourth reaction step, and (e) fifth reaction step.

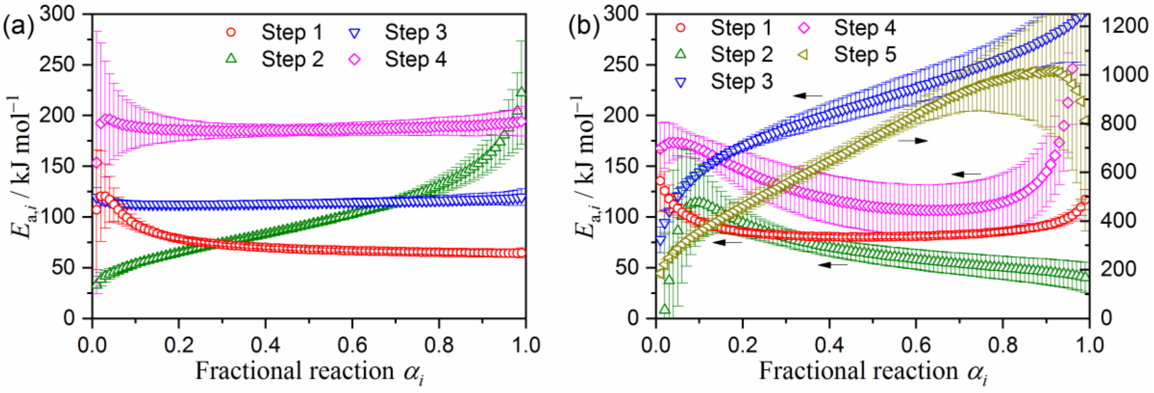

Figure S16. E_a values at different α_i for each reaction step i of the multistep thermal decomposition for the clay mineral substrates: (a) palygorskite and (b) sepiolite.

S3. Kinetic analysis for the thermal decomposition of Maya blue

Table S2 lists the contributions c_i of each separated reaction step that was calculated based on the ratio of the mathematically separated peak areas . The sums of the contributions from the additional peaks were 0.41 and 0.28 in the P-MB and S-MB samples, respectively. As an alternative outcome from MDA, a series of the kinetic curves for each reaction step at different β were generated, as illustrated in Figures S17 and S18 for the P-MB and S-MB, respectively. The kinetics curves were subjected to isoconversional kinetic analysis using the Friedman plot. Changes in $E_{a,i}$ for each reaction step as the reaction advanced in each reaction step are illustrated in Figure S19 by selecting the reaction steps attributable to the thermal decomposition of indigo in the MB samples. Table S2 also lists the $E_{a,i}$ values for each reaction step averaged over the restricted α_i ranges, where the values were relatively constant. in. In comparison with the $E_{a,i}$ values for the reaction steps in the thermal decomposition of the clay mineral substrates as determined by KDA (Table 1) for palygorskite and sepiolite the average $E_{a,i}$ values for the corresponding reaction steps attributed to the thermal decomposition of the respective clay mineral substrates in the thermal decomposition of the MB samples were approximately the same, except for the third reaction step in the thermal decomposition of S-MB.

Table S2. Contributions c_i of each reaction step i to the total mass-loss value for the overall thermal decomposition of the MB samples as determined by MDA and $E_{a,i}$ values, which have been determined by the Friedman plot applied to the kinetic curves for each individual reaction step separated by MDA

sample	step i	c_i	$E_{a,i}$ / kJ mol$^{-1,a}$
P-MB	1	0.09 ± 0.02	80.1 ± 2.3 ($0.1 \le \alpha_1 \le 0.6$)
	2	0.10 ± 0.01	78.2 ± 1.3 ($0.3 \le \alpha_2 \le 0.5$)
	3	0.10 ± 0.01	88.7 ± 1.5 ($0.2 \le \alpha_3 \le 0.9$)
	4	0.09 ± 0.01	53.3 ± 16.4 ($0.1 \le \alpha_4 \le 0.9$)
	5	0.15 ± 0.01	142.3 ± 5.2 ($0.1 \le \alpha_5 \le 0.9$)
	6	0.30 ± 0.02	212.0 ± 33.6 ($0.1 \le \alpha_6 \le 0.9$)
	7	0.12 ± 0.02	563.5 ± 72.6 ($0.1 \le \alpha_7 \le 0.5$)
	8	0.05± 0.02	431.9 ± 6.6 ($0.2 \le \alpha_8 \le 0.8$)
S-MB	1	0.15 ± 0.02	81.7 ± 4.4 ($0.1 \le \alpha_1 \le 0.5$)
	2	0.07 ± 0.01	45.9 ± 1.2 ($0.1 \le \alpha_2 \le 0.7$)
	3	0.13 ± 0.02	100.6 ± 10.5 ($0.2 \le \alpha_3 \le 0.9$)
	4	0.04 ± 0.02	163.6 ± 10.1 ($0.2 \le \alpha_4 \le 0.8$)
	5	0.34 ± 0.09	127.6 ± 9.3 ($0.1 \le \alpha_5 \le 0.3$)
	6	0.05 ± 0.04	180.4 ± 2.2 ($0.3 \le \alpha_6 \le 0.5$)
	7	0.07 ± 0.02	103.9 ± 15.2 ($0.2 \le \alpha_7 \le 0.8$)
	8	0.12 ± 0.03	284.8 ± 55.0 ($0.1 \le \alpha_8 \le 0.6$)
	9	0.03 ± 0.02	720.2 ± 52.6 ($0.1 \le \alpha_9 \le 0.2$)

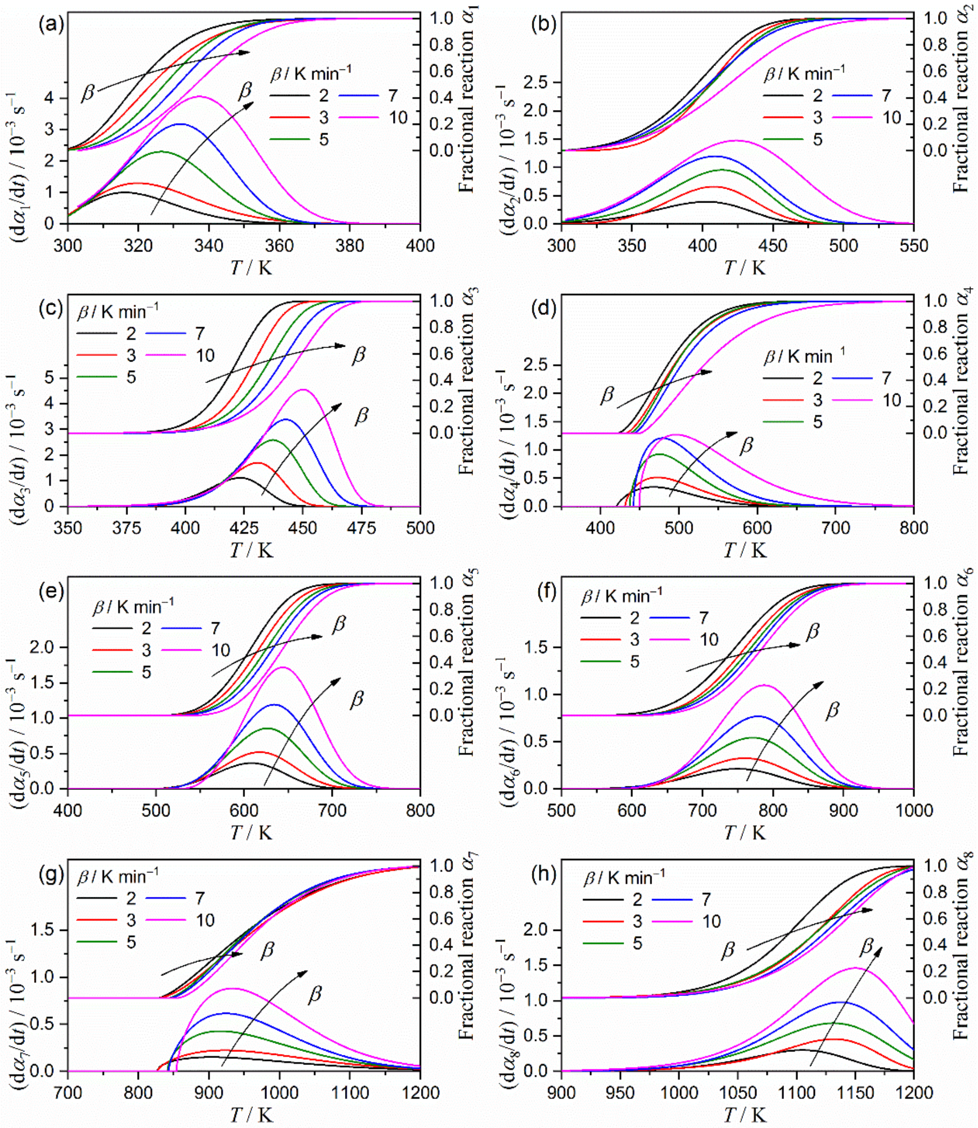

Figure S17. Kinetic curves for each reaction step of the multistep thermal decomposition of the P-MB sample, generated by MDA: (a) first, (b) second, (c) third, (d) fourth, (e) fifth, (f) sixth, (g) seventh, and (h) eighth reaction steps.

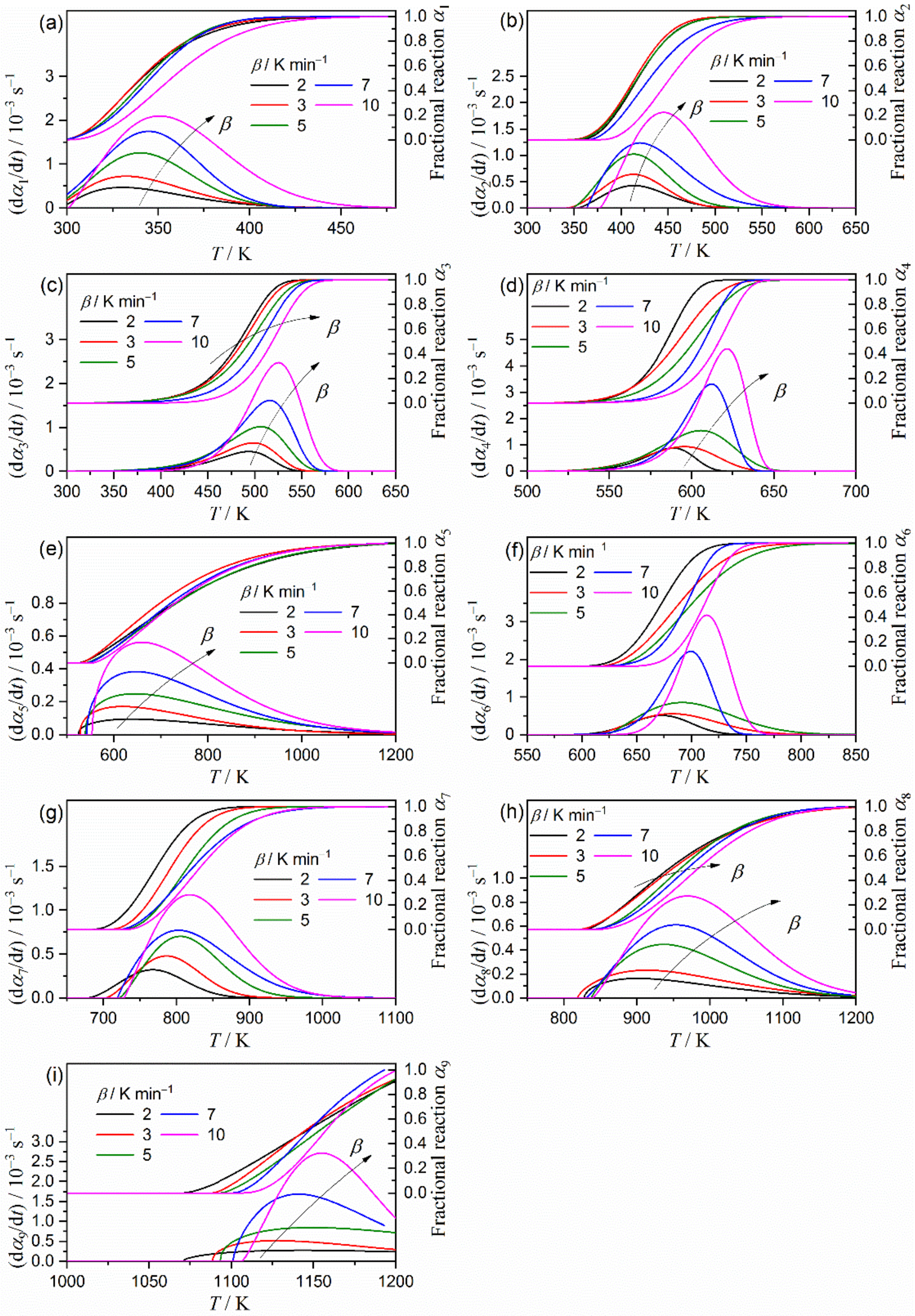

Figure S18. Kinetic curves for each reaction step of the multistep thermal decomposition of the S-MB sample, generated by MDA: (a) first, (b) second, (c) third, (d) fourth, (e) fifth, (f) sixth, (g) seventh, (h) eighth, and (i) ninth reaction steps.

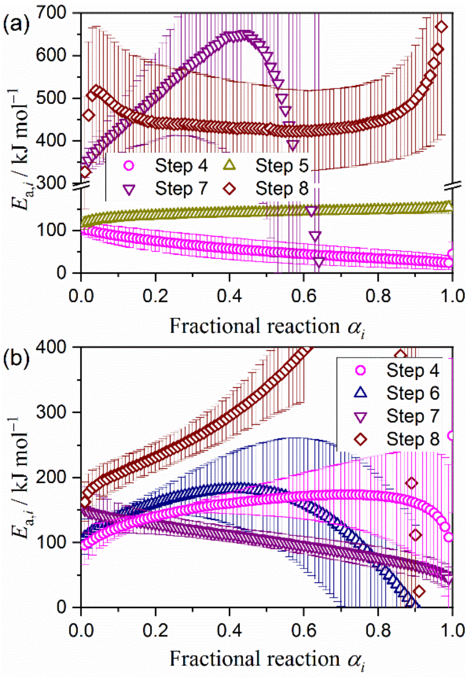

Figure S19. Changes in $E_{a,i}$ with α_i, as determined by the Friedman plot applied to the kinetic curves generated by MDA for the reaction steps attributed to the thermal decomposition of indigo in an MB sample: (a) P-MB and (b) S-MB samples.

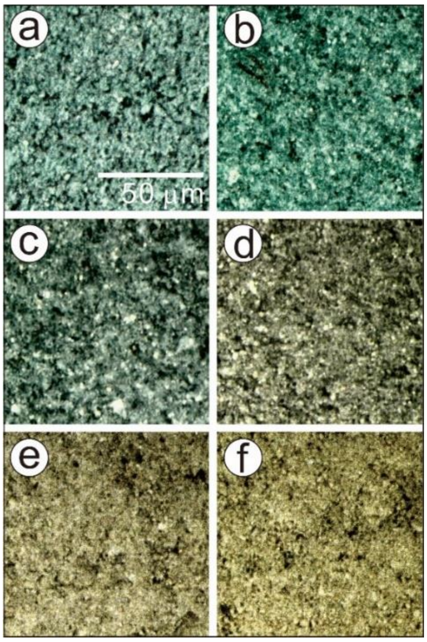

Figure S20. Typical optical microscopic views of S-MB when heated to different temperatures: (a) original sample, (b) 573 K, (c) 653 K, (d) 753 K, (e) 1003 K, and (f) 1093 K.

S4. Kinetic analysis for the thermally induced sublimation/decomposition of indigo

Figure S21 presents a series of TG–DTG curves of indigo reagent recorded at different β values in flowing air. The two-step mass loss was characteristic of this reaction. The second mass-loss process was interpreted as the oxidative decomposition of the decomposition product in the previous reaction step, because of the accompanying exothermic DTA peak. At the same time, significant deposition of indigo was observed in the cold part of the reaction tube during the measurement. Therefore, the first mass-loss process was interpreted as a combination of both indigo sublimation and decomposition. Figure S22 summarizes the results of the kinetic analysis for the first mass-loss process of the sublimation/decomposition of indigo. Friedman plots at each α value indicated a statistically significant linear correlation between $\ln(\mathrm{d}\alpha/\mathrm{d}t)$ and T^{-1}: the correlation coefficients γ were better than 0.99 in the range of $0.02 \le \alpha \le 0.92$ (Figure S22(a)). The slopes of the Friedman plots were approximately invariant during the first mass-loss process, thus obtaining an approximately constant E_a value (Figure S22(b)) with an average value of 150.7 ± 4.7 kJ mol^{-1} ($0.10 \le \alpha \le 0.90$). Based on this constant E_a value, the rate behavior of the first mass-loss process was reproduced at infinite temperature as the experimental master plot of $(\mathrm{d}\alpha/\mathrm{d}\theta)$ versus α, where the $(\mathrm{d}\alpha/\mathrm{d}\theta)$ value at a selected α was calculated according to Eq. (S2) [S9-S11]:

$$\frac{\mathrm{d}\alpha}{\mathrm{d}\theta} = \left(\frac{\mathrm{d}\alpha}{\mathrm{d}t}\right)\exp\left(\frac{E_a}{RT}\right)$$

$$\text{with} \quad \theta = \int_0^t \exp\left(-\frac{E_a}{RT}\right)\mathrm{d}t \qquad \text{(S2)}$$

where θ is Ozawa's generalized time [S12], which denotes hypothetical reaction time at infinite temperature. The experimental master plot indicated gradual deceleration of the reaction rate in a convex shape as the reaction advances (Figure S22(c)), which is explained by the shrinkage of indigo crystalline particles by sublimation from the surfaces and by phase boundary-controlled model.

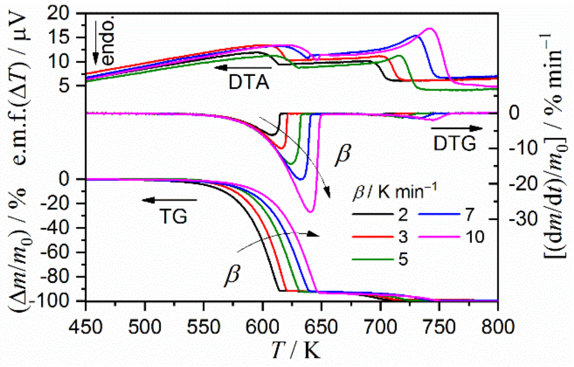

Figure S21. A series of TG–DTG–DTA curves for the indigo reagent (m_0 = 1.0 mg) recorded at different β values in flowing air (300 cm^3 min^{-1}).

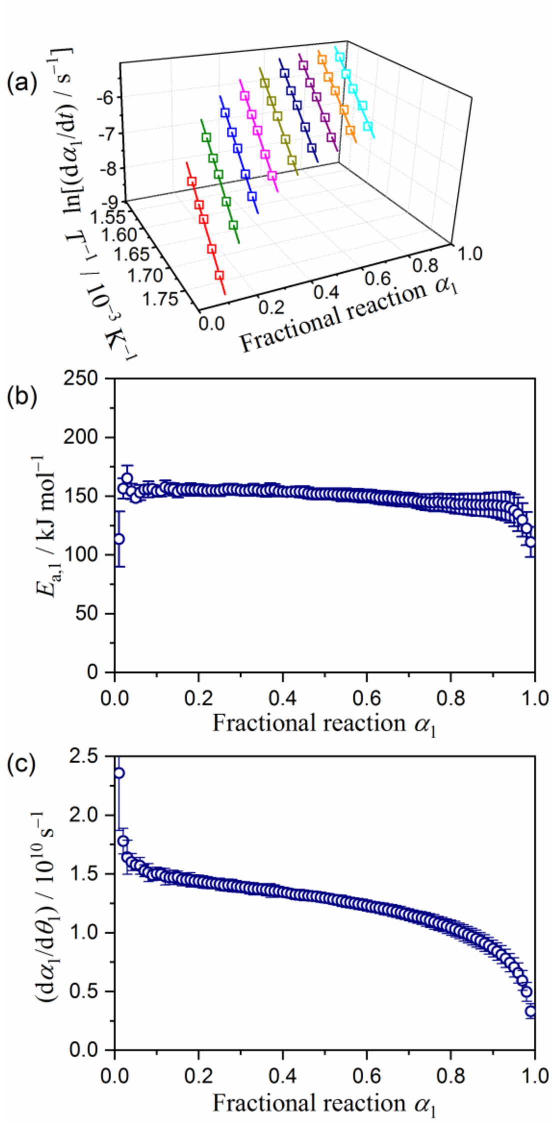

Figure S22. Typical result of the isoconversional kinetic analysis for the first mass-loss process of sublimation/decomposition of indigo reagent: (a) Friedman plots at different α values, (b) E_a value at different α values, and (c) experimental master plot of $(d\alpha_1/d\theta_1)$ versus α_1.

References

S1. Chiari, G.; Giustetto, R.; Ricchiardi, G., Crystal structure refinements of palygorskite and Maya blue from molecular modelling and powder synchrotron diffraction. *Eur. J. Mineral.* **2003,** 15, (1), 21-33. doi:10.1127/0935-1221/2003/0015-0021.

S2. Giustetto, R.; Chiari, G., Crystal structure refinement of palygorskite from neutron powder diffraction. *Eur. J. Mineral.* **2004,** 16, (3), 521-532. doi:10.1127/0935-1221/2004/0016/0521.

S3. Preisinger, A., X-ray study of the structure of sepiolite. *Clays Clay Miner.* **1959,** 6, (1), 61-67. doi:10.1346/CCMN.1957.0060106

S4. Andersen, F. A.; Brečević, L., Infrared spectra of amorphous and crystalline calcium carbonate. *Acta Chem. Scand.* **1991,** 45, 1018-1024. doi:10.3891/acta.chem.scand.45-1018.

S5. Vagenas, N. V.; Gatsouli, A.; Kontoyannis, C. G., Quantitative analysis of synthetic calcium carbonate polymorphs using FT-IR spectroscopy. *Talanta* **2003,** 59, (4), 831-836. doi:10.1016/S0039-9140(02)00638-0.

S6. Yan, W.; Liu, D.; Tan, D.; Yuan, P.; Chen, M., FTIR spectroscopy study of the structure changes of palygorskite under heating. *Spectrochim. Acta, A. Mol. Biomol. Spectrosc.* **2012,** 97, 1052-7. doi:10.1016/j.saa.2012.07.085.

S7. Alkan, M.; Benlikaya, R., Poly(vinyl alcohol) nanocomposites with sepiolite and heat-treated sepiolites. *J. Appl. Polym. Sci.* **2009,** 112, (6), 3764-3774. doi:10.1002/app.29830.

S8. Hayashi, H.; Otsuka, R.; Imai, N., Infrared study of sepiolite and palygorskite on heating. *Am. Mineral.* **1969,** 54, (11-12), 1613-1624.

S9. Ozawa, T., A new method of analyzing thermogravimetric data. *Bull. Chem. Soc. Jpn.* **1965,** 38, (11), 1881-6. doi:10.1246/bcsj.38.1881.

S10. Koga, N., Kinetic analysis of thermoanalytical data by extrapolating to infinite temperature. *Thermochim. Acta* **1995,** 258, 145-159. doi:Doi 10.1016/0040-6031(95)02249-2.

S11. Gotor, F. J.; Criado, J. M.; Málek, J.; Koga, N., Kinetic analysis of solid-state reactions: The universality of master plots for analyzing isothermal and nonisothermal experiments. *J. Phys. Chem. A* **2000,** 104, (46), 10777-10782. doi:10.1021/jp0022205.

S12. Ozawa, T., Non-isothermal kinetics and generalized time. *Thermochim. Acta* **1986,** 100, (1), 109-18. doi:10.1016/0040-6031(86)87053-8.
